# Liver-specific Nr1h4 deletion in mice with human-like bile acid composition causes severe liver injury

**DOI:** 10.1016/j.jlr.2025.100839

**Published:** 2025-06-09

**Authors:** Yusuke Mishima, Kota Tsuruya, Kinuyo Ida, Satsuki Ieda, Yutaka Inagaki, Akira Honda, Tatehiro Kagawa, Akihide Kamiya

**Affiliations:** 1 Division of Gastroenterology and Hepatology, Department of Internal Medicine, Tokai University School of Medicine, Isehara, Kanagawa, Japan; 2 Department of Molecular Life Sciences, Tokai University School of Medicine, Isehara, Kanagawa, Japan; 3 Center for Matrix Biology and Medicine, Tokai University School of Medicine, Isehara, Kanagawa, Japan; 4 Joint Research Center, Tokyo Medical University Ibaraki Medical Center, Ami, Ibaraki, Japan; 5 Department of Gastroenterology and Hepatology, Tokyo Medical University Ibaraki Medical Center, Ibaraki, Japan

**Keywords:** nuclear receptor

## Abstract

The farnesoid X receptor, encoded by *NR1H4*, is crucial for bile acid, lipid, and glucose metabolism. *NR1H4* mutations in humans cause a severe liver injury called progressive familial intrahepatic cholestasis 5. However, Nr1h4 deletion in mice did not cause severe liver damage at a young age, likely because of the higher levels of hydrophilic bile acids synthesized by the mouse-specific bile acid metabolic enzymes Cyp2a12 and Cyp2c70. We aimed to assess hepatic NR1H4 function by taking advantage of the recently established Cyp2a12/Cyp2c70 double-knockout (CYPDKO) mouse model, which has a human-like bile acid composition containing mainly hydrophobic bile acids. Liver-specific Nr1h4-deficient CYPDKO mice were established using an adeno-associated virus-derived genome-editing method. Nr1h4-deficient wild-type (WT) mice showed no significant changes in marker levels for serum liver injury. In contrast, Nr1h4-deficient CYPDKO mice showed an increase in the liver/body weight ratio and serum liver injury markers, suggesting that the combination of human-like bile acid composition and Nr1h4 deletion induces liver injury. Nr1h4 deletion increased total bile acid levels in the liver through the upregulation of bile acid metabolic genes and downregulation of bile acid transporters. Conversely, overexpression of a small heterodimer partner (Shp), a downstream gene of Nr1h4, suppresses liver injury induced by Nr1h4 deletion in CYPDKO mice. Overall, liver-specific Nr1h4 deficiency induced significant liver damage in mice with human-like bile acids, unlike in WT mice, validating its use as a new animal model for cholestatic liver disease. Therefore, Shp may be a potential target for the treatment of cholestasis.

The liver is the largest metabolic organ in the body and plays an important role in serum protein synthesis, drug metabolism, and lipid, cholesterol, and bile acid synthesis. Bile secretion is one of the most important liver functions. Bile secreted by hepatocytes is stored in the gallbladder and excreted in the small intestine. In the small intestine, bile causes micellization of lipids in food for digestion. Bile is a mixture of bile acids, phospholipids, bilirubin, and cholesterol. Primary bile acids are synthesized from cholesterol in hepatocytes and are secreted into the small intestine via the gallbladder. Secondary bile acids are mostly absorbed in the large intestine after they are initially synthesized from primary bile acid by microbial enzymes. Nuclear receptors are a family of transcription factors that regulate gene expression upon binding to ligands such as steroids and hormones (1). Various types of nuclear receptors exist, ranging from NR0 to NR6, each of which controls the function of various organs, including the liver. NR1H4 encodes the farnesoid X receptor (FXR), a nuclear receptor that is mainly expressed in the liver and small intestine and is activated by binding to bile acids, particularly chenodeoxycholic acid (CDCA), with high affinity (2, 3). It regulates cholesterol and lipid metabolism. For example, NR1H4 in the small intestine regulates the expression of fibroblast growth factor (FGF) 19 (FGF 15 in rodents) (4, 5), which is secreted from small intestinal cells upon bile acid stimulation and acts on hepatocytes via the portal vein to control the expression of bile acid synthesis enzymes, such as CYP7A1 (6). FXR expressed in hepatocytes also regulates the expression of CYP7A1 and CYP8B1 by regulating the expression of downstream transcription factors such as SHP (NR0B2). Nr1h4 gene deletion in mice elevated serum and liver levels of cholesterol and triglycerides and increased the cholic acid pool size in association with increased expression of Cyp7a1 and Cyp8b1 (7, 8). Therefore, NR1H4 is a central regulator of lipid, cholesterol, and bile acid metabolism.

Feedback mechanisms strictly regulate bile secretion and composition. Some bile acids are highly hydrophobic, supporting lipid digestion, but are cytotoxic at high concentrations. Therefore, dysfunction of bile acid metabolism and transport systems leads to liver injury and diseases, such as cholestasis (9, 10). ABCB11 and ABCB4 are transporters that export bile acids and phospholipids from hepatocytes to the bile canaliculus, respectively, and loss-of-function mutations in these transporters cause progressive familial intrahepatic cholestasis (PFIC) (11). PFIC-related gene knockout mice have been developed as models for analyzing PFIC symptoms in vivo. However, mice lacking Abcb11 or Abcb4 do not show severe liver injury, as observed in human PFIC (12, 13). This finding might be due to differences in bile acid composition between humans and mice. Rodents have unique bile acid metabolic enzymes that humans lack: Cyp2a12 converts secondary bile acids, lithocholic acid (LCA), and deoxycholic acid (DCA) to primary bile acids, and Cyp2c70 converts CDCA to muricholic acid (MCA). These enzymes metabolize hydrophobic bile acids into hydrophilic bile acids. Recently developed Cyp2c70/Cyp2a12 double-knockout (CYPDKO) mice have a humanized hydrophobic bile acid composition (14). Previously, we constructed a new PFIC3 pathological model by inducing Abcb4 deficiency in CYPDKO mice using genome editing with adeno-associated viruses (AAV) (15). Compared to normal mice with Abcb4 deficiency, these mice showed elevated serum liver injury markers, inflammatory cytokines, and humoral factors, along with pseudo-bile duct proliferation, inflammatory cell infiltration, and fibrosis promotion in liver histology. These results suggest that human-like bile acid composition is critical for the reproduction of cholestasis.

In this study, we focused on the role of NR1H4 in bile acid metabolism under human-like bile acid conditions. A recent study demonstrated that human NR1H4 mutations induce cholestasis and hepatocyte death by suppressing ABCB11 expression (16). Although Nr1h4-deficient mice also showed decreased Abcb11 expression and elevated serum cholesterol and triglyceride levels, liver injury was mild over a short period, indicating that Nr1h4 deficiency in conventional mice may not replicate human PFIC (7). Therefore, we induced liver-specific Nr1h4 deficiency in CYPDKO mice and analyzed its effects on liver pathology. CYPDKO/Nr1h4-knockout (KO) mice showed elevated serum levels of liver injury markers, inflammatory cytokines, and fibrosis-related genes, which were not observed in the wild-type (WT)/Nr1h4-KO mice. Furthermore, overexpression of Shp, a downstream transcriptional regulator of Nr1h4, in CYPDKO/Nr1h4-KO mice ameliorated the Nr1h4-deficiency-induced liver injury. This study revealed the importance of the hepatic NR1H4-SHP axis in cholestatic liver injury under human-like bile acid composition.

## Materials and methods

### Experimental animals

**Fig. 1:**
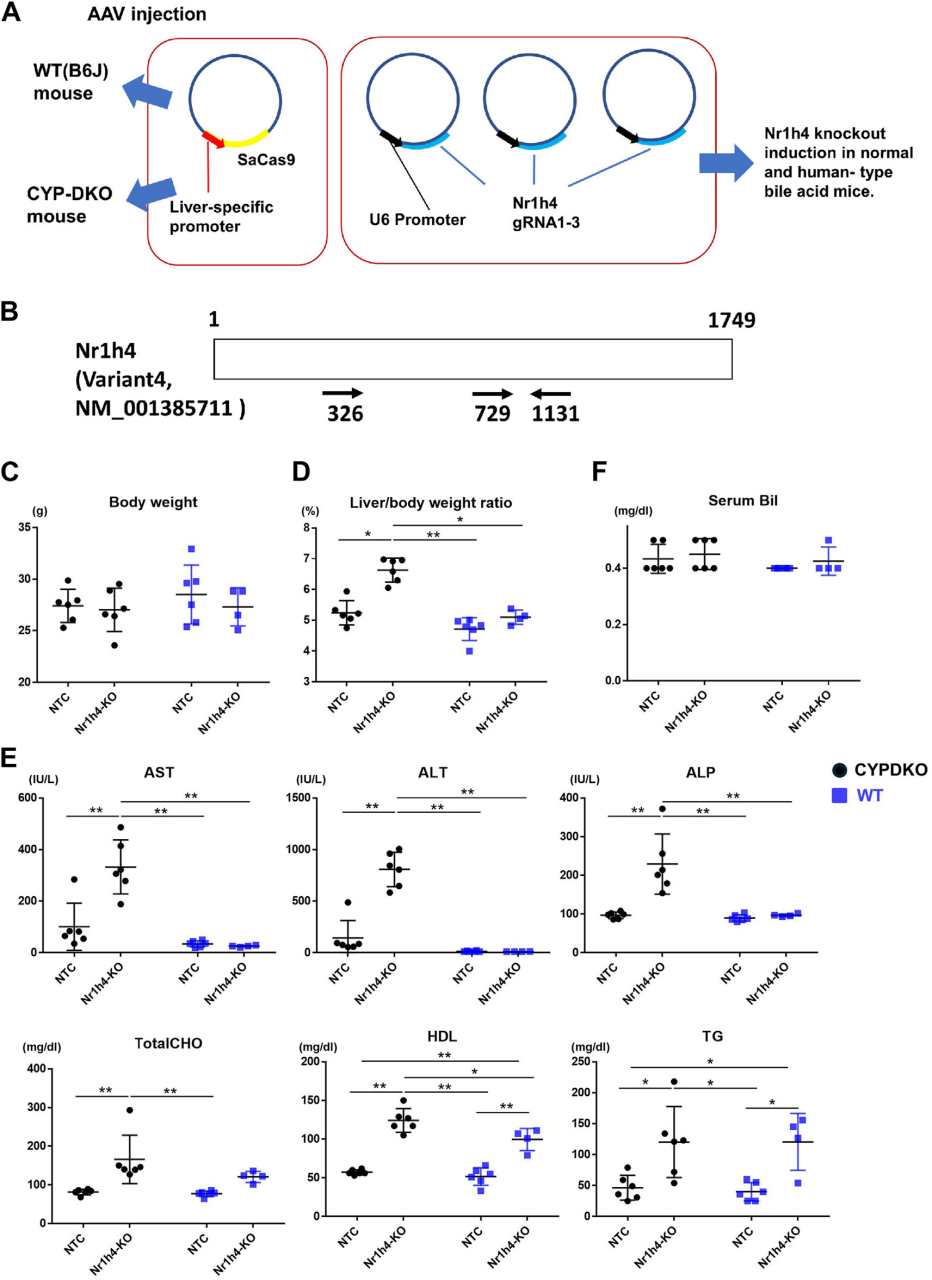
Effect of Nr1h4 deficiency in a human-like bile acid model using a liver-specific AAV-CRISPR method. A: Generation of liver-specific Nr1h4-deletion mice infected with an AAV expressing SaCas9 and specific gRNAs. B: Three gRNAs targeting Nr1h4 were used. AAVs expressing gRNAs that targeted the indicated sites in the CDS region were generated. C: Changes in body weight. D: Changes in the liver/body weight ratio. E: Changes in serum liver injury marker (AST, ALT, and ALP), total cholesterol (TotalCHO), HDL, and triglyceride (TG) levels. F: Changes in serum bilirubin (Bil). Nr1h4gRNA-transduced WT and CYODKO mice were analyzed as WT/Nr1h4-knockout (KO) and CYPDKO/Nr1h4-KO mice (n = 6 for CYPDKO/NTC, n = 6 for CYPDKO/Nr1h4-KO, n = 6 for WT/NTC, and n = 4 for WT/Nr1h4-KO). Results are presented as mean ± SD (two-way ANOVA, ∗*P* < 0.05, ∗∗*P* < 0.01).

C57BL6/J mice were purchased from Nihon SLC and CLEA Japan Inc. CYPDKO mice have been previously described (14). Mice were maintained on a 12-h dark/light cycle with free access to food (CA-1, a Good Laboratory Practice-compliant diet for the breeding of inbred animals, CLEA Japan, Inc) and water, as shown previously (15). For AAV injection, 10-13-week-old CYPDKO and C57BL6/J mice were used. For sample collection, almost 4–6 weeks after AAV injection, the mice were euthanized between 12:00 and 16:00 under isoflurane anesthesia (15). Male CYPDKO and C57BL6/J mice were used because of spontaneous liver injury in female CYPDKO mice (14). In the experiment to analyze Nr1h4-defect in CYPDKO and WT mice (Fig. 1), mice (aged 81.0–83.3 days) were injected with AAV, and samples were collected, averaging 38.1–38.5 days later. In the experiment to analyze Shp overexpression in CYPDKO/Nr1h4-KO mice, mice (aged 79.0–79.9 days) were injected with AAV, and samples were collected, averaging 31.4–32.2 days later. All animal experimental protocols were approved by the Institutional Animal Care and Use Committee of Tokai University (approval numbers 243010, 232,006, 232,008, 221,011, and 221,105).

### Virus construction

AAV vectors for in vivo genome editing were constructed as described previously (15). Briefly, the SaCas9 expression vector and human-U6 promoter-regulated guide RNA (gRNA) expression vectors were transfected into HEK293T cells with the AAV8 capsid (Penn Vector Core, University of Pennsylvania) and helper plasmids (Takara Bio Inc.) (17). The rAAV2-LSP1 vector was used for the liver-specific overexpression of Shp (18). This AAV vector has apolipoprotein E enhancer and human alpha-antitrypsin promoter for liver-specific expression. AAV was purified from cells and concentrated using an AAVPro Purification Kit (Takara Bio Inc.). AAV titers were measured using the AAVpro Titration Kit Ver2 (Takara Bio, Inc.). The gRNA sequences were designed using CRISPOR (http://crispor.tefor.net/). The target sequences of the gRNA are listed in Supplemental Table S1.

### AAV injection into mice for gene deletion

After anesthesia was induced through isoflurane inhalation via the oral cavity, AAVs expressing SaCas9 in combination with either enhanced green fluorescent protein (*Egfp*) gRNA (negative control) or three types of gRNAs for target genes were administered intraperitoneally (2.0–3.0 × 10^11^ vg/mouse). To delete *Nr1h4*, CYPDKO, and C57BL/6J male mice were used. Mice infected with AAVs expressing SaCas9 and *Egfp*-gRNA or mice without AAVs were used as negative controls (NTC). For Shp overexpression in CYPDKO/Nr1h4-KO mice, AAVs expressing SaCas9, three types of gRNA, and AAV expressing Shp were administered. To select mice for AAV injection with either the *Egfp* control gRNA or target gene gRNAs, mice of approximately the same weight were randomly selected. One CYPDKO and one CYPDKO/Nr1h4 mouse were excluded from the analyses due to liver atrophy caused by unknown reasons.

### Gene expression analyses using quantitative RT-PCR

Immediately after collection, the liver and small intestine (ileum) tissues were soaked in RNAlater solution (Thermo Fisher Scientific), stored overnight at 4 °C, placed in RNAiso Plus (Takara Bio Inc.), and homogenized using ShakeMaster NEO (Bio Medical Science Inc) with stainless beads (15). Chloroform was added, and the solution was centrifuged, after which the supernatant was separated, and RNA was extracted by isopropanol precipitation. First-strand cDNA for quantitative PCR was synthesized from 0.5 μg of RNA using ReverTra Ace qPCR RT Master Mix with gDNA Remover (TOYOBO). The target gene expression was corrected for the TATA-binding protein. Quantitative analysis of the target mRNA was performed using a universal probe library system (Roche Diagnostics, Basel, Switzerland) and either the TAMRA-FAM probe (Takara Bio, Inc.) or SYBR PCR. Primers and probes used are listed in Supplemental Table S2.

### Analysis of cholesterol, phospholipid, and bile acid levels in the gallbladder and small intestine

The small intestines were solubilized in 1 M NaOH at 80 °C for 20 min. The biliary gallbladders of mice were surgically dissected and collected into 1.5 ml tubes. After the gallbladder bile was diluted with 150 μl distilled water, cholesterol, and phospholipid concentrations were determined in the gallbladder bile using LabAssay Kits (FUJIFILM Wako Pure Chemical). Total bile acid levels in the gallbladder bile and small intestine were analyzed using a Total Bile Acid Assay Kit (CBL-STA631, Cell Biolabs, Inc.) (15).

### Analyses of bile acid composition in the liver

The liver samples were solubilized in 1 M NaOH/water at 80 °C for 20 min. After the addition of internal standards and 0.5 mol/L potassium phosphate buffer (pH 7.4), bile acids were extracted and quantified by Liquid Chromatography-Mass Spectrometry. The mass analysis protocol has been previously described (14). Hydrophobicity indices were calculated using individual bile acid data from a previous study (15).

### Statistical analysis

Unpaired *t* test, one-way ANOVA with Tukey's multiple comparisons test, two-way ANOVA with Sidak's multiple comparisons test, and Kruskal–Wallis tests with Dunn's multiple comparisons test were performed using Prism7 (GraphPad Software), and SDs were calculated, and statistically significant differences were determined.

All other methods are described in the Supporting Materials and Supplemental Tables S1 and S2.

## Results

### Induction of liver injury by liver-specific Nr1h4 deficiency using AAV

Liver-specific gene deficiency was induced using the in vivo CRISPR method, in which human α1-antitrypsin promoter-dependent SaCas9-expressing AAV and target gene gRNA-expressing AAV were simultaneously infected (15). Using this system, we generated liver-specific Nr1h4-deficient WT and CYPDKO mice (Fig. 1A and B). We manufactured AAVs expressing three target gRNAs against exon regions common to each variant of Nr1h4 using the human U6 promoter. These gRNA expression vectors were introduced into WT (C57BL/6J) and CYPDKO mice together with SaCas9 expression AAVs. Hepatic FXR protein levels were attenuated in WT and CYPDKO mice that received Nr1h4 gRNA (Supplemental Figure S1). We compared these four mouse groups (WT/NTC, WT/Nr1h4-KO, CYPDKO/NTC, and CYPDKO/Nr1h4-KO). Nr1h4-deficient WT (WT/Nr1h4-KO) mice did not show significant changes in body weight, liver weight ratio, or liver injury marker levels (Fig. 1C–E). However, serum cholesterol and triglyceride levels increased compared to those in WT/NTC mice, similar to previous results in conventional Nr1h4-KO mice, indicating an important role of NR1H4 in lipid metabolism (7). CYPDKO mice have a hydrophobic bile acid composition, and mild liver damage may occur; however, liver injury induced by Cyp2a12 and Cyp2c70 deletion is not significant in male mice (14). Nr1h4-deficient CYPDKO (CYPDKO/Nr1h4-KO) mice showed an increase in the liver/body weight ratio and serum liver injury markers such as alanine aminotransferase (ALT), aspartate aminotransferase (AST), and alkaline phosphatase (ALP), suggesting that hepatic Nr1h4-deficiency evokes severe liver injury only under human-like bile acid composition. In contrast, no change was observed in the serum bilirubin levels under any condition (Fig. 1F).

**Fig. 2:**
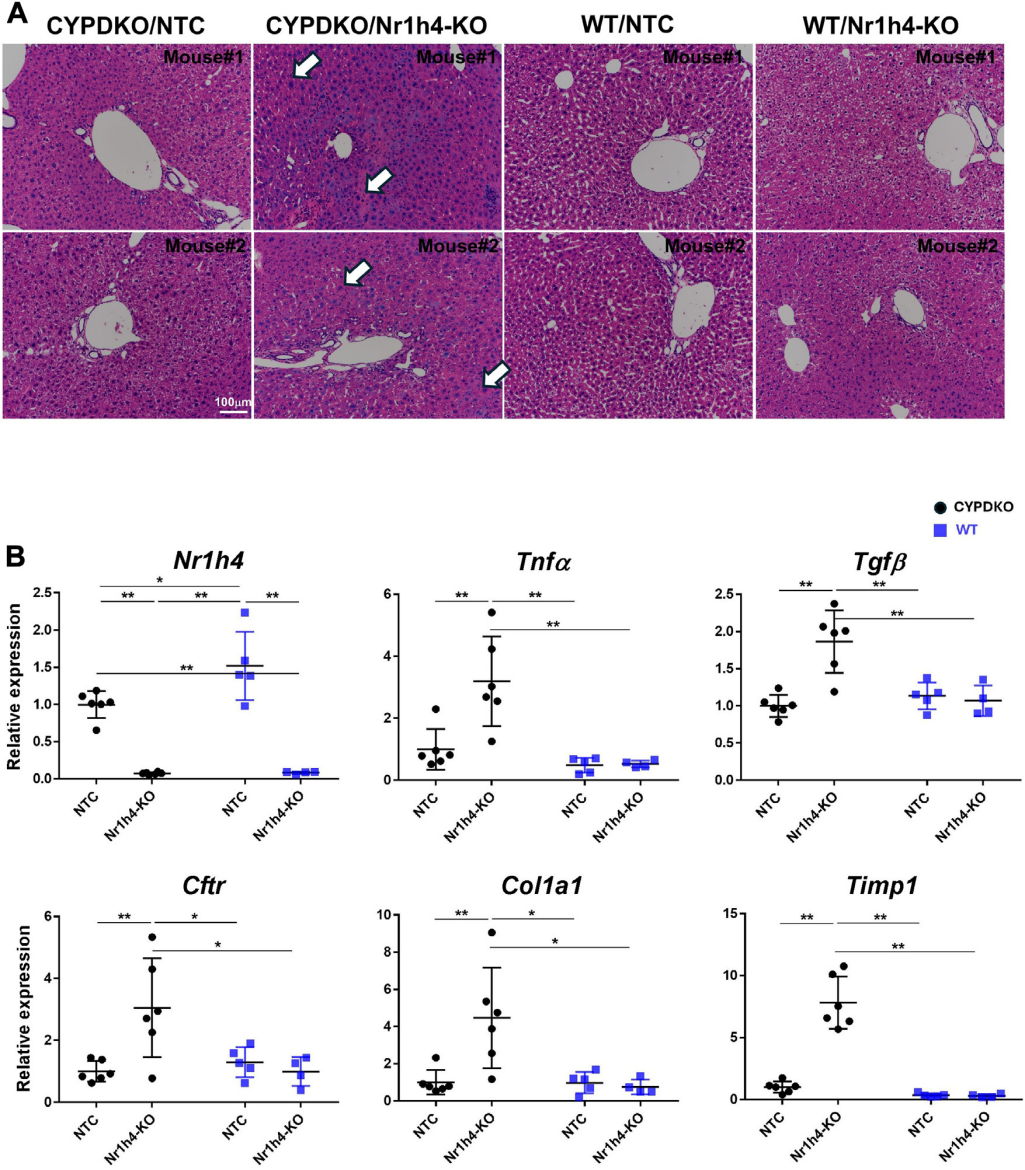
Liver injury and inflammation in CYPDKO/Nr1h4-deficient mice. A: Changes in hepatocyte morphology induced by the Nr1h4 deletion. Hematoxylin and eosin-stained sections derived from two individual CYPDKO/NTC, CYPDKO/Nr1h4-KO, WT/NTC, and WT/Nr1h4-KO mouse livers are shown. Arrows show enlarged hepatocytes and disorganized structures of the mature hepatocyte cords. White line, 100 μm. B: Changes in gene expression of inflammatory and fibrotic markers in the liver. (n = livers for CYPDKO/NTC mice was set to 1.0 (n = 6 for CYPDKO/NTC, n = 6 for CYPDKO/Nr1h4-KO, n = 5 for WT/NTC, and n = 4 for WT/Nr1h4-KO). Results are presented as mean ± SD (two-way ANOVA, ∗*P* < 0.05, ∗∗*P* < 0.01).

Liver tissue sections from CYPDKO/Nr1h4-KO mice showed enlarged hepatocytes and disorganized structures of mature hepatocytic cords, suggesting inflammation (Fig. 2A). The expression of inflammatory cytokines and fibrosis markers, such as tumor necrosis factor (Tnf) α, transforming growth factor (Tgf) β, collagen 1a1, and Timp1, in the liver was elevated only in CYPDKO/Nr1h4-KO mice (Fig. 2B). In contrast, bile ductal cell proliferation, which was previously found in CYPDKO/Abcb4-KO mice (15), was not observed (Supplemental Figure S2).

### Changes in bile acid metabolism in liver-specific Nr1h4 deficiency

**Fig. 3:**
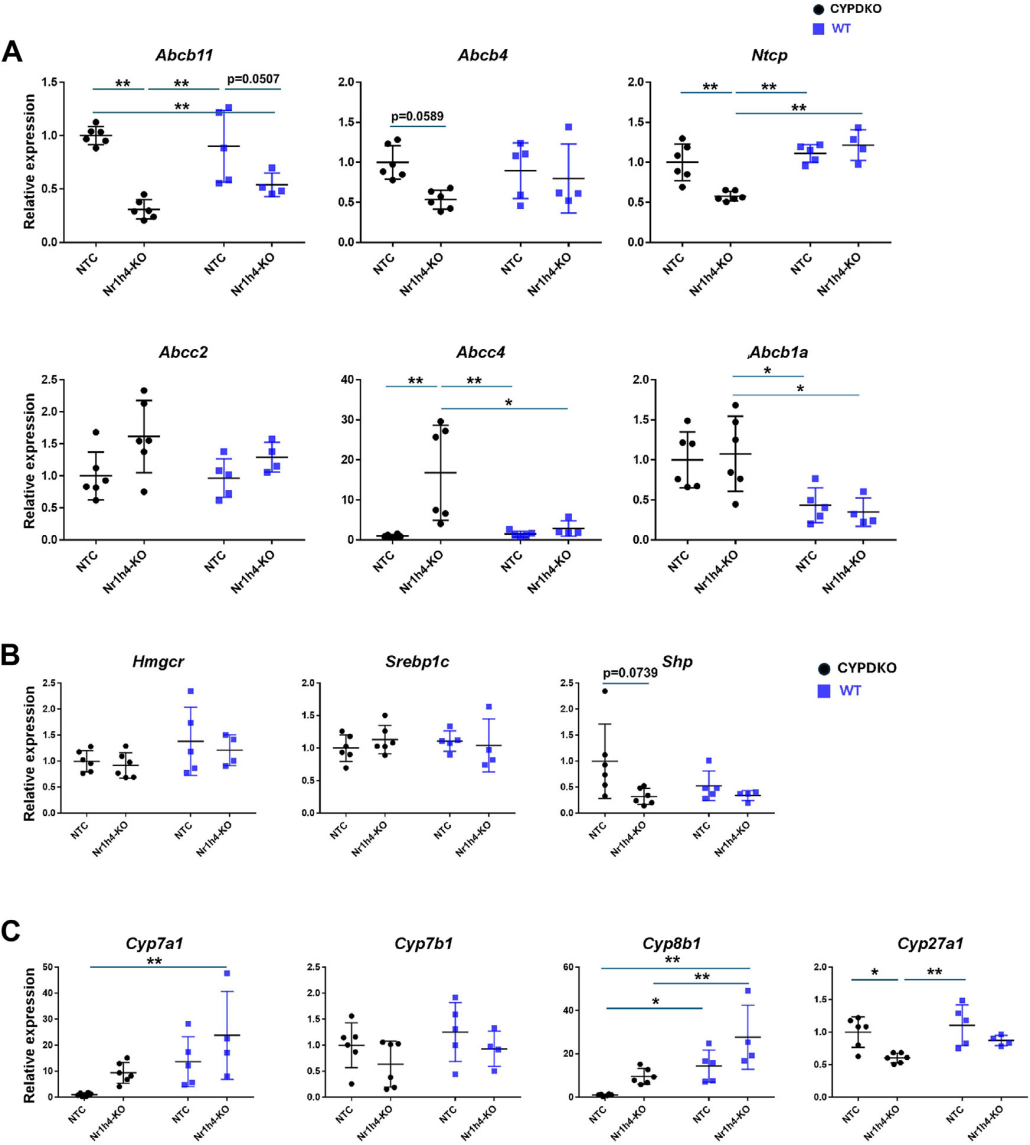
Lipid and bile acid metabolic changes in the livers of CYPDKO/Nr1h4-deficient mice. A–C: Gene expression changes in membrane transporters (A), lipid and bile acid metabolic genes (B), and glucose metabolic genes (C) in the liver. The expression of genes in the livers of CYPDKO/NTC mice was set to 1.0 (n = 6 for CYPDKO/NTC, n = 6 for CYPDKO/Nr1h4-KO, n = 5 for WT/NTC, and n = 4 for WT/Nr1h4-KO). Results are presented as mean ± SD (two-way ANOVA, ∗*P* < 0.05, ∗∗*P* < 0.01).

NR1H4 deficiency in humans may cause liver injury by reducing ABCB11 expression in hepatocytes (16). Therefore, we analyzed the changes in the hepatic expression of bile transporters and metabolism-related genes in Nr1h4-KO mice (Fig. 3A). Abcb11 expression tended to be reduced by Nr1h4-deficiency in WT and CYPDKO mice. In addition, the expression of Ntcp was reduced specifically in CYPDKO/Nr1h4-KO mice compared to other mouse groups, indicating that the transport function of bile acids was reduced. Increased expression of Abcc4 may represent an adaptive mechanism against liver injury, as this transporter plays a role in the efflux of excess bile acids from hepatocytes into the blood. Expression of cholesterol and lipid metabolism-related genes Hmgcr and Srebp1c was not changed by Nr1h4-deletion in both WT and CYPDKO mice (Fig. 3B). In CYPDKO/NTC mice, Cyp7a1 and Cyp8b1 expression was suppressed, similar to a previous report (14), compared to that in WT mice, probably because of the feedback mechanism that inhibits further bile acid synthesis by large amounts of NR1H4-ligand hydrophobic bile acids induced by Cyp2a12/2c70 deletion (Fig. 3C). Thus, we compared the expression of bile metabolic genes in the presence or absence of Nr1h4 under human-like bile acid conditions. Hepatic Nr1h4 deletion in CYPDKO mice abrogated Cyp7a1 and Cyp8b1 suppression. In contrast, the expression of Cyp27a1 was suppressed by Nr1h4 deletion in CYPDKO mice (Supplemental Figure S3).

### Suppression of liver injury by Shp overexpression in liver-specific Nr1h4-deficient mice

**Fig. 4:**
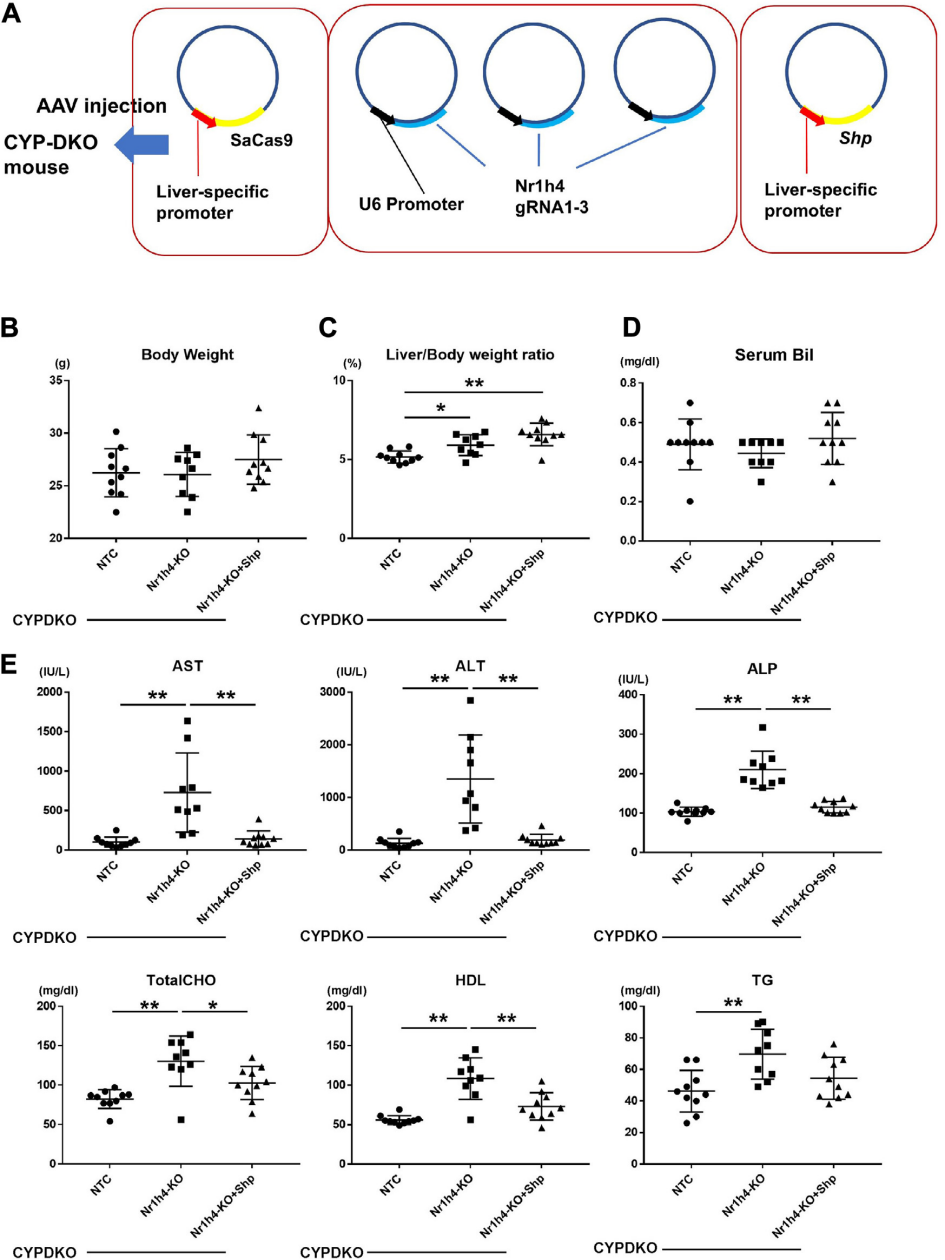
Shp-overexpression compensated for Nr1h4-deficiency-induced liver injury. A: Shp expression in CYPDKO/liver-specific Nr1h4-deletion mice following AAV infection. B: Changes in body weight. C: Changes in the liver/body weight ratio. D: Changes in serum bilirubin (Bil). E: Changes in serum liver injury marker (AST, ALT, and ALP), total cholesterol (TotalCHO), HDL, and triglyceride (TG) levels. (n = 10 for CYPDKO/NTC, n = 9 for CYPDKO/Nr1h4-KO, and n = 10 for CYPDKO/Nr1h4-KO + Shp). Results are presented as mean ± SD (one-way ANOVA, ∗*P* < 0.05, ∗∗*P* < 0.01).

The combination of hepatic Nr1h4-deficiency and humanization of the bile acid composition alters the metabolism and transport of lipids and bile acids, leading to liver inflammation. However, the underlying mechanisms remain unknown. NR1H4 suppresses Cyp7a1 and Cyp8b1 expression by inducing SHP expression (19, 20). To analyze whether Shp is involved in metabolic abnormalities and liver injury in CYPDKO/Nr1h4-KO mice, we performed a rescue experiment using the liver-specific overexpression of Shp in CYPDKO-KO mice (Fig. 4A). The liver/body weight ratio elevated in CYPDKO/Nr1h4-KO mice was not affected by Shp induction (Fig. 4B and C). No changes were observed in the serum bilirubin levels in any mouse group (Fig. 4D). In contrast, the increase in serum liver injury markers such as AST, ALT, and ALP, as well as serum cholesterol levels, was blunted by Shp overexpression in CYPDKO/Nr1h4-KO mice (Fig. 4E). Therefore, Shp signaling suppresses liver injury caused by Nr1h4 deficiency under human-like bile acid conditions.

**Fig. 5:**
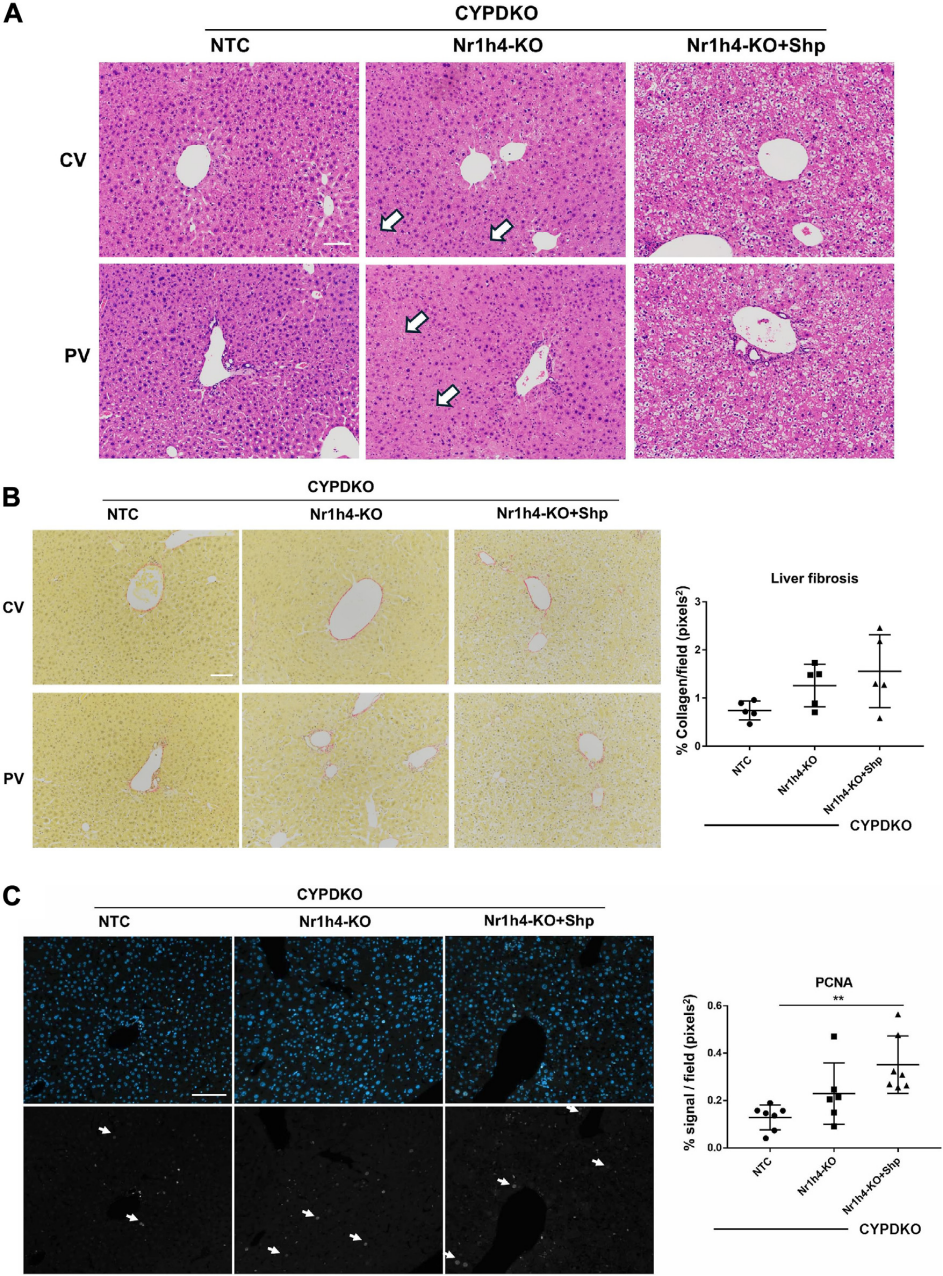
Effect of Shp on inflammation and fibrosis in CYPDKO/Nr1h4-deficient mice. A: Changes in the hepatocyte morphology. Hematoxylin and Eosin staining was performed. Arrows indicate enlarged hepatocytes and disorganized structures of the mature hepatocytic cords. B: Analysis of intrahepatic fibrosis using Sirius red staining. (B, *right panel*) Sirius red staining was performed to visualize liver fibers and quantify the number of liver fibers using ImageJ software (n = 5 for the livers of CYPDKO/NTC, CYPDKO/Nr1h4-KO, and CYPDKO/Nr1h4-KO + Shp mice). C: Analysis of cell proliferation using PCNA staining. (C, *right panel*) PCNA expression levels were determined by measuring liver cell proliferation using the ImageJ software (n = 7 for the livers of CYPDKO/NTC, n = 6 for CYPDKO/Nr1h4-KO, and n = 7 for CYPDKO/Nr1h4-KO + Shp mice). CV, central vein; PV, portal vein. White line, 100 μm. Results are presented as mean ± SD (one-way ANOVA, ∗∗*P* < 0.01).

Enlarged hepatocytes and disorganized structures induced by Nr1h4 deficiency were not detected in CYPDKO/Nr1h4-KO mice overexpressing Shp (Fig. 5A and S4A). To analyze cell death induced by Nr1h4 deficiency, we detected apoptotic cells using TUNEL staining of mouse liver sections. A small number of TUNEL-positive cells were observed in CYPDKO/Nr1h4-KO mice, whereas almost no TUNEL-positive cells were observed under other conditions (Supplemental Figure S4B and C). These results suggest that Nr1h4 deficiency induces liver injury mediated by apoptosis, although only in part, and this effect is suppressed by Shp.

Kupffer cell and macrophage marker F4/80 staining of the liver sections showed that the number of Kupffer cells and macrophages tended to be higher in CYPDKO/Nr1h4-KO mice than in CYPDKO/NTC mice (*P* = 0.0513, Supplemental Figure S5A and B). In contrast, bile ductal cell proliferation was unchanged by liver-specific Nr1h4 deficiency (Supplemental Figure S5C and D). We analyzed the induction of fibrosis by Nr1h4-deficiency using Sirius red staining (Fig. 5B). Nr1h4 deficiency in CYPDKO mice induced slight, but not significant, accumulation of collagen fibers, which was not affected by Shp overexpression. Cell proliferation in the injured liver was analyzed using proliferating cell nuclear antigen (PCNA) staining (Fig. 5C). However, significant cell proliferation was not detected in CYPDKO/Nr1h4-KO mice. In contrast, CYPDKO/Nr1h4-KO + Shp mice had more proliferative cells than CYPDKO/NTC mice did.

**Fig. 6:**
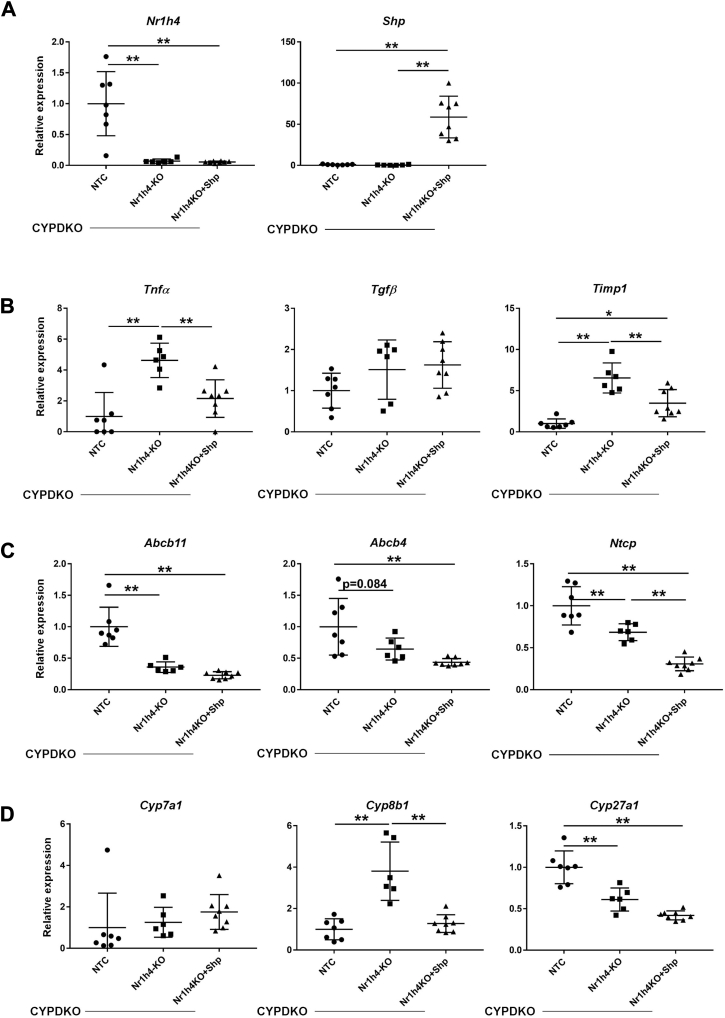
Nr1h4-deficiency-induced inflammation and lipid metabolic changes compensated by Shp overexpression. A–D: Gene expression changes in Nr1h4 and Shp (A), inflammatory and fibrotic markers (B), bile transporters (C), and bile acid metabolic genes (D) in the liver. The expression of genes in CYPDKO/NTC mouse livers was set to 1.0 (n = 7 for CYPDKO/NTC livers, n = 6 for CYPDKO/Nr1h4-KO, and n = 8 for CYPDKO/Nr1h4-KO + Shp mice). Results are represented as mean ± SD (one-way ANOVA, ∗*P* < 0.05, ∗∗*P* < 0.01).

Next, we analyzed the changes in the expression of inflammation and fibrosis markers, as well as bile acid and lipid metabolism-related genes. Hepatic deletion of Nr1h4 and overexpression of Shp were confirmed by RT-PCR (Fig. 6A). Nr1h4-deficiency in CYPDKO mice increased inflammatory and fibrosis markers, such as Tnfα, Il1β, Ccl2, and Timp1 (Fig. 6B and S6A). Some inflammatory cytokines and Timp1 were suppressed by Shp overexpression. The decrease in Abcb11 and Ntcp expression caused by Nr1h4-deficiency was not reversed by Shp overexpression (Fig. 6C). In particular, the amount of the Abcb11-coding protein Bsep was analyzed in CYPDKO/NTC and CYPDKO/Nr1h4-KO mouse livers using immunofluorescence histochemistry. The deletion of Nr1h4 significantly suppressed the Bsep protein level, and this downregulation was not recovered by overexpression of Shp (Supplementl Figure S7). Expression of Abcc4 was induced in CYPDKO/Nr1h4-KO mice but not in CYPDKO/Nr1h4-KO + Shp mice (Supplemental Figure S6B), indicating that this was consistent with reduced inflammation. The expression of Cyp7a1 did not change between CYPDKO/Nr1h4-KO mice with and without Shp overexpression. In contrast, the upregulation of Cyp8b1 caused by Nr1h4-deficiency was abolished by Shp overexpression, whereas the reduction in Cyp27a1 expression was unaffected (Fig. 6D). In addition, we analyzed the expression of Nr1h4-related genes in the terminal ileum; however, no significant changes were observed in Fgf15, Abst, and iBabp (Supplemental Figure S6C). These results suggest that Shp overexpression ameliorates liver injury by regulating bile acid synthesis and transport and inhibiting cellular inflammation.

### Changes in bile acid metabolism in liver-specific Nr1h4-deficiency under human-like bile acid composition

**Fig. 7:**
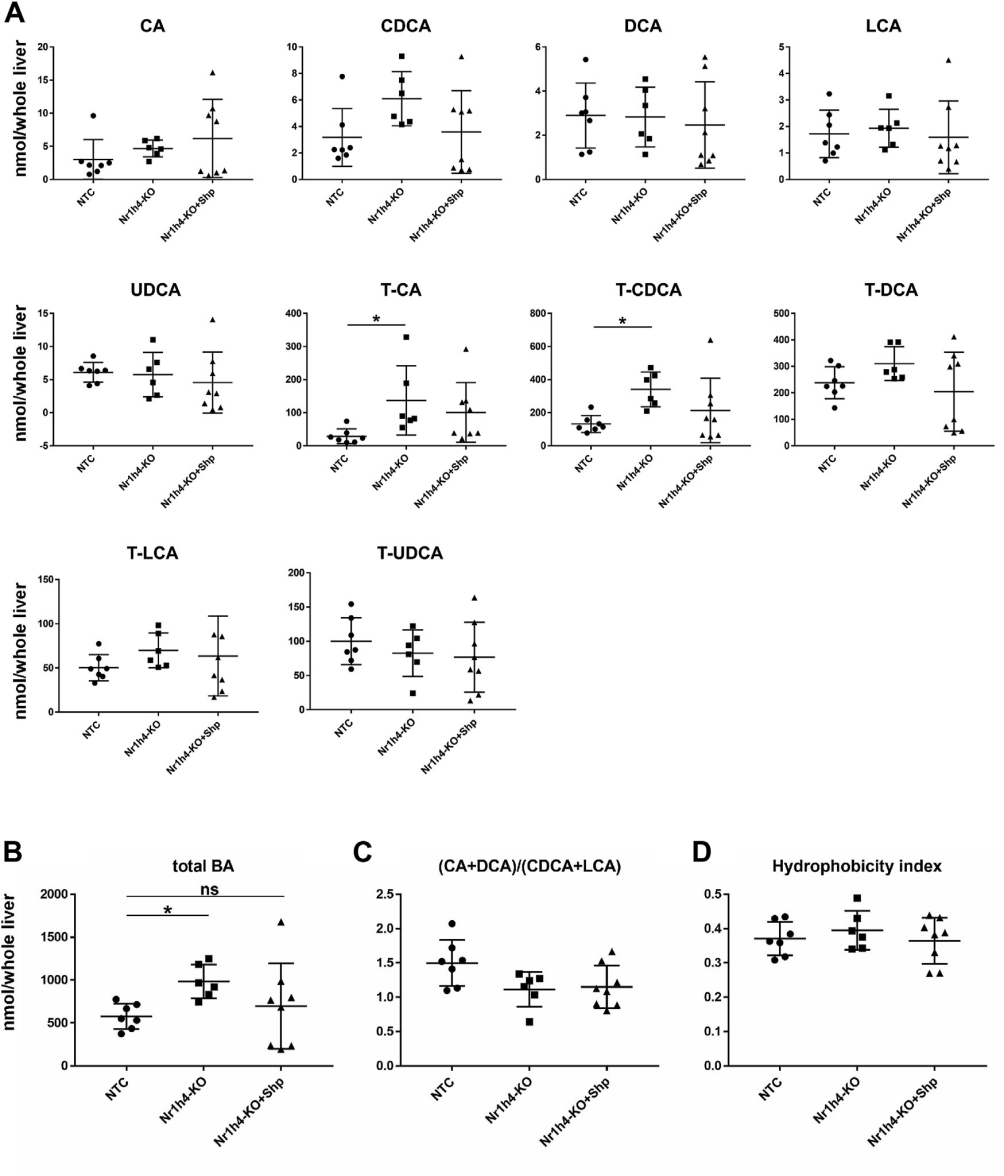
Effect of Shp on bile acid composition in the livers of CYPDKO/Nr1h4-deficient mice. A and B: Levels of various total bile acids in the liver of CYPDKO mice with Nr1h44 deficiency and Shp overexpression. C: The (CA + DCA)/(CDCA + LCA) ratio of bile acids in the livers of Nr1h4-deleted CYPDKO mice. D: Hydrophobicity indices of total bile acids in the liver. The hydrophobicity index was calculated as the percentage-weighted mean of the individual bile acid hydrophobicity (n = 7 for the livers of CYPDKO/NTC, n = 6 for CYPDKO/Nr1h4-KO, and n = 8 for CYPDKO/Nr1h4-KO + Shp mice). Results are presented as the mean ± SD (Kruskal–Wallis tests, ∗*P* < 0.05, ∗∗*P* < 0.01).

To analyze the changes in hepatic bile acid composition due to Nr1h4-deficiency, we analyzed bile acid levels in CYPDKO/NTC and CYPDKO/Nr1h4-KO mice (Supplemental Table S3 and Fig. 7A and B). As expected, MCAs were undetectable owing to a deficiency in mouse-specific Cyp2a12 and 2c70 (14). The total bile acid content in the liver was significantly higher in CYPDKO/Nr1h4-KO mice than in CYPDKO/NTC mice, with a significant increase in the taurine-conjugated CA (T-CA) and CDCA (T-CDCA) levels. The (CA + DCA)/(CDCA + MCA + LCA) ratio and hydrophobicity indices of hepatic bile acids did not differ among the three mouse groups (Fig. 7C and D). We also analyzed serum and gallbladder bile acid, phospholipid, and cholesterol levels; however, no significant changes were observed (Supplemental Figure S8A and B).

Next, we compared the amount of bile acids in the gallbladder, liver, and small intestine and the total bile pool (combined with the gallbladder, liver, and small intestine) in the whole body. Consistent with the results shown in Fig. 7B, Nr1h4 deficiency increased the amount of total bile acids in the liver (Supplemental Figure S8C). In contrast, there was no change in the amount of bile acids in the total bile pool of Nr1h4 deficiency. By overexpressing Shp in this Nr1h4 deficiency state, an increase in bile acid levels in the liver was not significantly observed, but the small intestine and the amount of bile acids in the total bile pool were decreased (Fig. 7B and S8C). This result is consistent with the results of a previous study (21). These results suggest that although the hydrophobicity (cytotoxicity) of the bile acid composition did not change under Nr1h4 deficiency in CYPDKO mice, Nr1h4 deficiency showed liver-specific cholestasis and induced liver damage. On the other hand, overexpression of Shp in CYPDKO/Nr1h4-KO mice reduced the total amount of bile pool in the whole body, suggesting its involvement in suppressing liver damage.

## Discussion

Conventional Nr1h4-KO mice lacking Nr1h4 expression in all organs show slightly elevated serum bile acid concentrations and serum and liver triglyceride levels (7, 8). Nr1h4-KO young mice exhibit normal body weight and behavior similar to that of WT mice (22). Severe cholestasis was barely observed in young male mice with Nr1h4-deficiency. In contrast, hepatic lipid accumulation and an increase in serum liver injury markers, bile acids, and bilirubin were observed in aged mice, which exhibit symptoms of steatohepatitis and cholestasis, as well as the development of hepatocellular carcinomas (23, 24). Although PFIC5 associated with human NR1H4-deficiency causes severe liver injury accompanied by cholestasis at a young age, mouse Nr1h4-deficiency is almost normal at a young age, and metabolic diseases such as steatohepatitis caused by abnormalities in lipid metabolism could underlie liver injury in aged mice. In this study, WT mice with Nr1h4-deficiency induced by AAV-mediated genome editing showed increased serum cholesterol and triglyceride levels, whereas no liver damage was observed, indicating that Nr1h4-deficiency alone did not induce short-term liver injury in mice, unlike in humans. In contrast, the combination of human-like bile acid composition and Nr1h4 deficiency causes severe liver injury, hepatocyte abnormalities, and increased expression of inflammatory cytokines. These results indicate that hydrophobic bile acid composition is critical for creating cholestatic mouse models.

It remains unclear how differences in bile acid composition between mice and humans affect the pathology of cholestasis. It was previously reported that conventional Nr1h4-KO mice did not show short-term liver injury with a normal diet. In contrast, a diet containing cholic acid, a hydrophobic bile acid, significantly increased hepatic bile acids and serum liver injury markers in addition to liver cell death. The cholic acid-containing diet activated NR1H4 by increasing hydrophobic bile acids, and downstream signaling induced by NR1H4 reduced the toxicity of cholic acid partly by activating the bile acid transport system through the induction of hepatocytic ABCB11 expression. In Nr1h4-KO mice, this compensatory mechanism does not function, leading to severe liver injury (7, 25). In CYPDKO mice, the ratio of hydrophobic bile acids in hepatocytes increases because of the lack of MCA synthesis (14). Thus, it was suggested that Nr1h4 deletion in mice with human-like bile acid composition induced liver injury because the compensatory mechanism responding to the change in bile acid composition did not work similarly to that in cholic acid-diet-fed Nr1h4-KO mice. Since Nr1h4-deficient mice fed a cholic acid diet showed liver damage through excessive oxidative stress, such oxidative stress stimuli might also be involved in manifesting the PFIC phenotype, and controlling oxidative stress is expected to be a future therapeutic option (26). Abcb11 expression levels were significantly lower in CYPDKO/Nr1h4-KO mice than in CYPDKO/NTC mice. This observation can be attributed to the lack of an NR1H4-mediated feedback mechanism against hydrophobic bile acids. The suppression of Ntcp expression in CYPDKO/Nr1h4-KO mice is unexpected. Ntcp mRNA expression is downregulated via the NR1H4-SHP pathway in response to excess bile acids (27). Therefore, Nr1h4-deficiency is expected to lose its feedback suppressive effects, resulting in increased Ntcp expression compared with that in CYPDKO/NTC mice. This discrepancy may be explained by the fact that inflammatory cytokines also repress Ntcp gene transcription, including Tnfα (28), which is elevated in CYPDKO/Nr1h4-KO mice.

In contrast to previous conventional Nr1h4-KO mouse studies, serum bile acid levels in CYPDKO/Nr1h4 mice did not change significantly, whereas bile acid levels in the entire liver increased. Our study differs from previous studies in that Nr1h4 expression was deleted in adult mice. The expression of Cyp7a1 and Cyp8b1 was not significantly altered in liver-specific Nr1h4-KO mice with normal hydrophilic bile acid composition (29). In contrast, our study revealed that Cyp7a1 and Cyp8b1 expressions tended to be repressed in a human-like bile acid mouse model and that liver-specific Nr1h4 deletion blunted the suppression of these genes. In conventional mice, hepatic Nr1h4 is constantly deactivated by the hydrophilic bile acid environment; therefore, deletion of hepatic Nr1h4 does not markedly alter bile acid metabolism. However, in CYPDKO mice, in which Nr1h4 is activated, as in humans, the loss of hepatic Nr1h4 significantly affects the bile acid metabolism. These results suggest that human-like bile acid composition might sufficiently activate NR1H4 to inhibit further bile acid synthesis in mice, and our mouse model helps study Nr1h4 metabolic functions under human-like bile acid conditions.

SHP, a nuclear receptor induced by NR1H4, plays an important role in Cyp7a1 and Cyp8b1 expression (20). In addition, Nr1h4/Shp double-KO mice, unlike Nr1h4 or Shp single-KO mice, developed severe cholestasis at an early stage, such as at three weeks of age (30). These results indicate that even in the absence of NR1H4, residual SHP can compensate for bile acid metabolism in the liver. A recent study showed that Shp overexpression suppresses the occurrence of hepatocellular carcinoma in aged Nh1h4-KO mice, partly by reducing serum bile acid and IL-6 levels (31). Analyses using Shp-transgenic mice have shown that Shp regulates the expression of genes involved in cholesterol degradation, bile acid conjugation, transport, and lipogenic pathways (32). Sustained Shp expression decreases the bile acid pool (21). In this study, we investigated whether Shp was involved in liver injury induced by CYPDKO/Nh1h4-KO mice. Analyses of bile acid pools in the liver, gallbladder, and small intestine indicated that the amount of bile acid in the liver was higher in CYPDKO/Nr1h4-KO mice than in CYPDKO/NTC mice. The quantitative PCR results suggested that the downregulation of Abcb11, regulation of hepatic bile acid secretion, and upregulation of Cyp8b1, which regulates the classic bile acid synthetic pathway, might be related to liver bile acid accumulation. In contrast, an increase in the amount of liver bile acid was not induced in CYPDKO/Nr1h4-KO + Shp mice compared with CYPDKO/NTC mice (a significant difference was not detected between the liver bile acid levels of CYPDKO/NTC and CYPDKO/Nr1h4-KO + Shp mice). Expression of hepatic bile acid secretion, Abcb11, did not change between CYPDKO/Nr1h4-KO and CYPDKO/Nr1h4-KO + Shp mice. However, Ntcp, which regulates hepatic bile acid intake, was significantly suppressed in CYPDKO/Nr1h4-KO + Shp mice compared to CYPDKO/Nr1h4-KO mice. Cyp8b1 expression, which was elevated in CYPDKO/Nh1h4-KO mice, was repressed by Shp overexpression, while Cyp7a1 expression remained unaffected. This observation is consistent with a previous study demonstrating that the repression of Cyp8b1 is more dependent on the presence of hepatic NR1H4/SHP than on Cyp7a1 (4). Interestingly, the bile acid levels in the small intestine and total bile pool were significantly decreased in CYPDKO/Nr1h4-KO + Shp mice compared to those in control mice. Thus, downregulation of the total bile acid pool might be important for the suppression of liver cholestasis injury.

In the present study, the increase in serum cholesterol and triglyceride levels induced by Nr1h4 deletion was attenuated by Shp overexpression. Shp regulates lipid metabolism and liver steatosis through NF-κB activation and Pparγ suppression (33). Additionally, several metabolic syndrome phenotypes were alleviated by Cyp8b1 deletion (34). Thus, various mechanisms regulated by Shp in bile acid and other lipid and cholesterol metabolism may play an important role in compensating for the predominance of hydrophobic bile acid composition. In this study, Shp overexpression in CYPDKO/Nr1h4-KO mice induced morphological changes and cell proliferation. The lipid and glucose metabolic changes induced by Shp may increase stress in Nr1h4-deficient hepatocytes. However, the results of serum liver injury marker, inflammatory gene expression, and bile acid pool analyses revealed that Shp partly compensated Nr1h4-induced hepatocyte malfunctions. Nr1h4 is expressed in the intestine and in addition to the liver and controls bile acid synthesis in the liver through the expression of the intestine Fgf15 and other factors. It is also involved in the reabsorption of bile acids in the intestine via Abst and other factors. However, in this study, Nr1h4 was deleted only in the liver, and no changes in the expression of Nr1h4-related genes in the intestine were observed. In this study, liver-only Nr1h4 deficiency-induced liver damage in combination with a hydrophobic bile acid composition demonstrated the importance of the bile acid metabolic system in humans centered on Nr1h4 in the liver. However, the role of Nr1h4 in the intestine remains unknown. In CYPDKO/Nh1h4-KO mice and human PFIC5, the regenerative mechanism against liver injury may be suppressed, making it a new target for pathological treatment. This PFIC5 mouse model with human-like bile acid composition is a promising tool for the study of human cholestatic diseases and the development of new drugs.

## Data availability

Data are available from the corresponding author upon reasonable request.

## Supplemental data

This article contains supplemental data.

## Conflict of interest

The authors declare that they have no conflicts of interest with the contents of this article.
