# Supplementary material for: Liver-specific Nr1h4 deletion in mice with human-like bile acid composition causes severe liver injury
Source: J Lipid Res. 2025 Jun 9;66(7):100839. doi: 10.1016/j.jlr.2025.100839 (PMC12332403; doi:10.1016/j.jlr.2025.100839)
Supplement: Supplementary Material 1 [file mmc1.pdf]

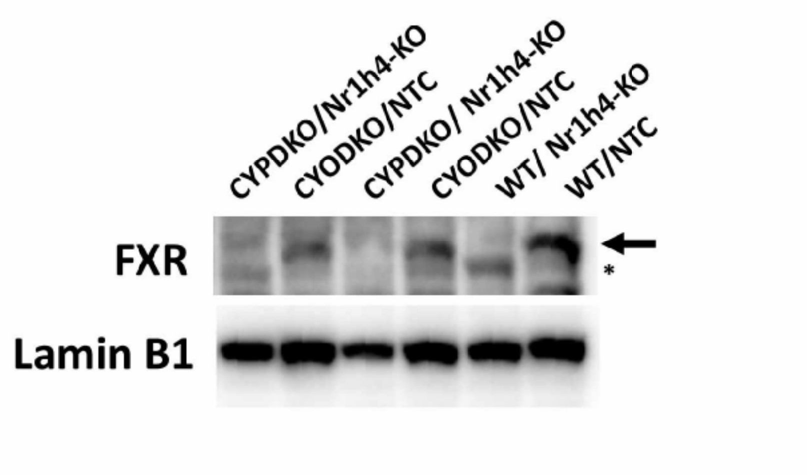

Figure S1 Expression of FXR protein regulated by AAV-mediated gene editing. After 4-5 weeks of AAV infection, the livers were analyzed using western blotting. The arrow shows the FXR protein and the asterisk shows nonspecific protein bands

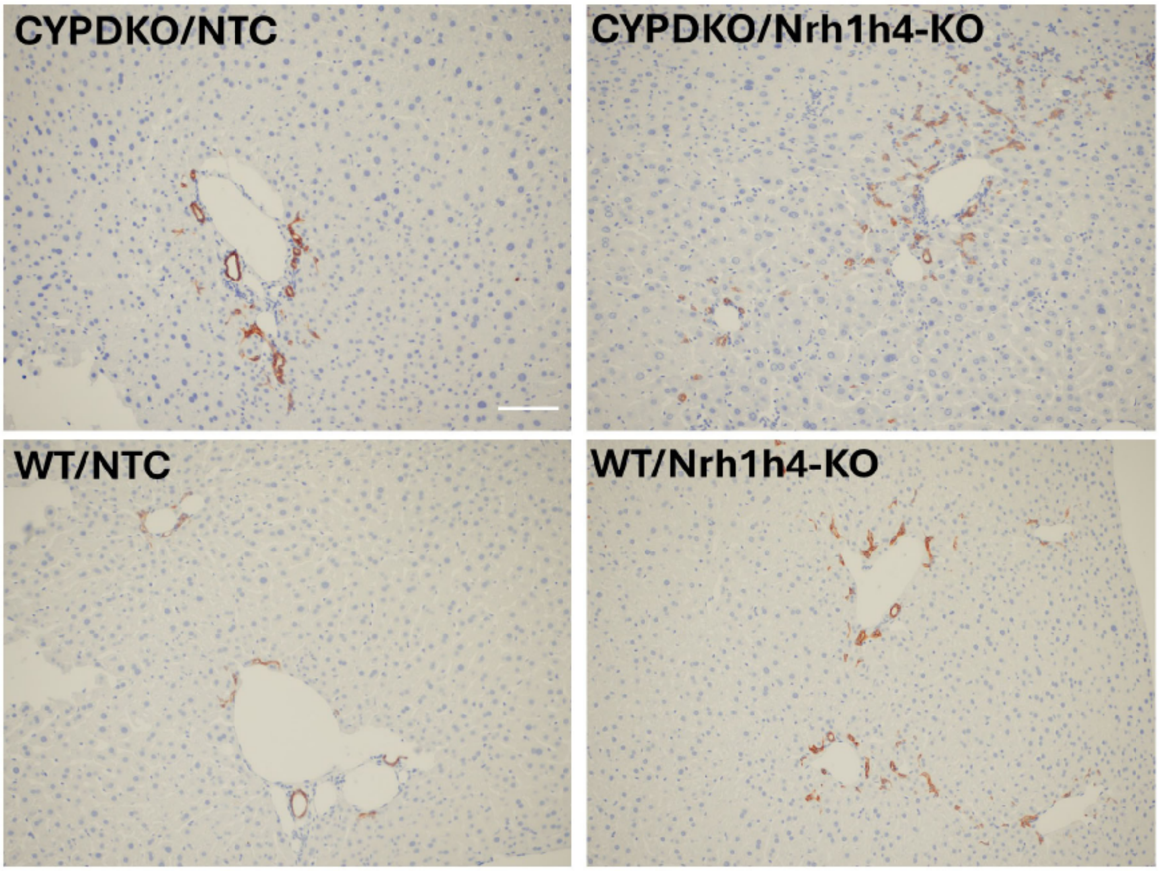

Figure S2 Bile duct cells in CYPDKO/Nr1h4-KO mouse livers. K19 immunostaining of CYPDKO and wild-type mice were performed. Nuclei were stained with hematoxylin. White line, 100 μm.

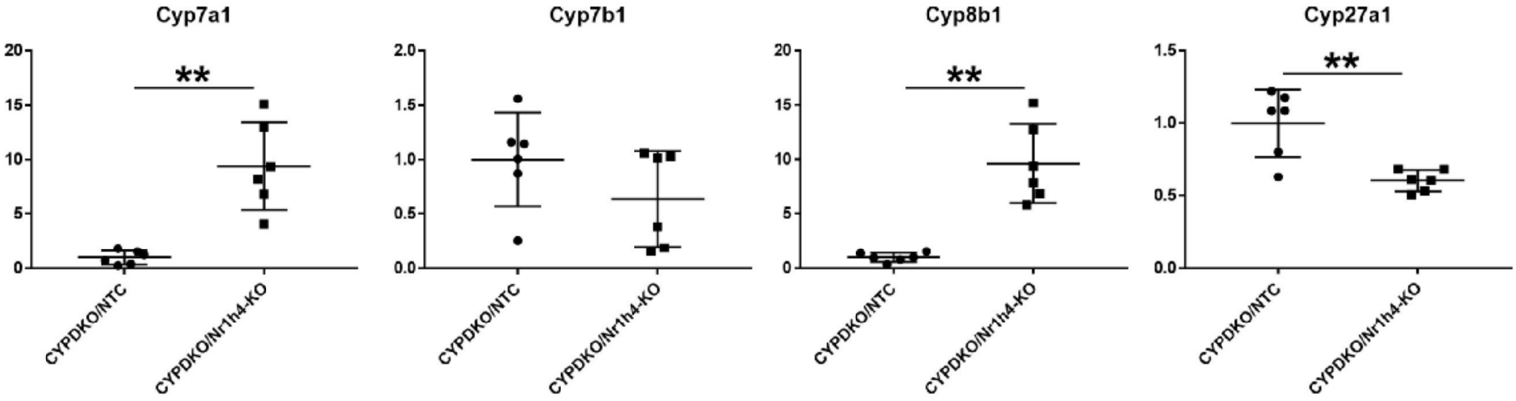

Figure S3 Bile acid metabolic gene expression changed by Nr1h4 knockout in the CYPDKO background. Gene expression changes in bile acid synthesis enzyme genes in the liver (n = 6 for CYPDKO/NTC and n = 6 for CYPDKO/Nr1h4-KO). The expression of CYPDKO/NTC in mice was set to 1.0. Results are presented as mean ± SD (unpaired *t* test, $^{**}P < 0.01$).

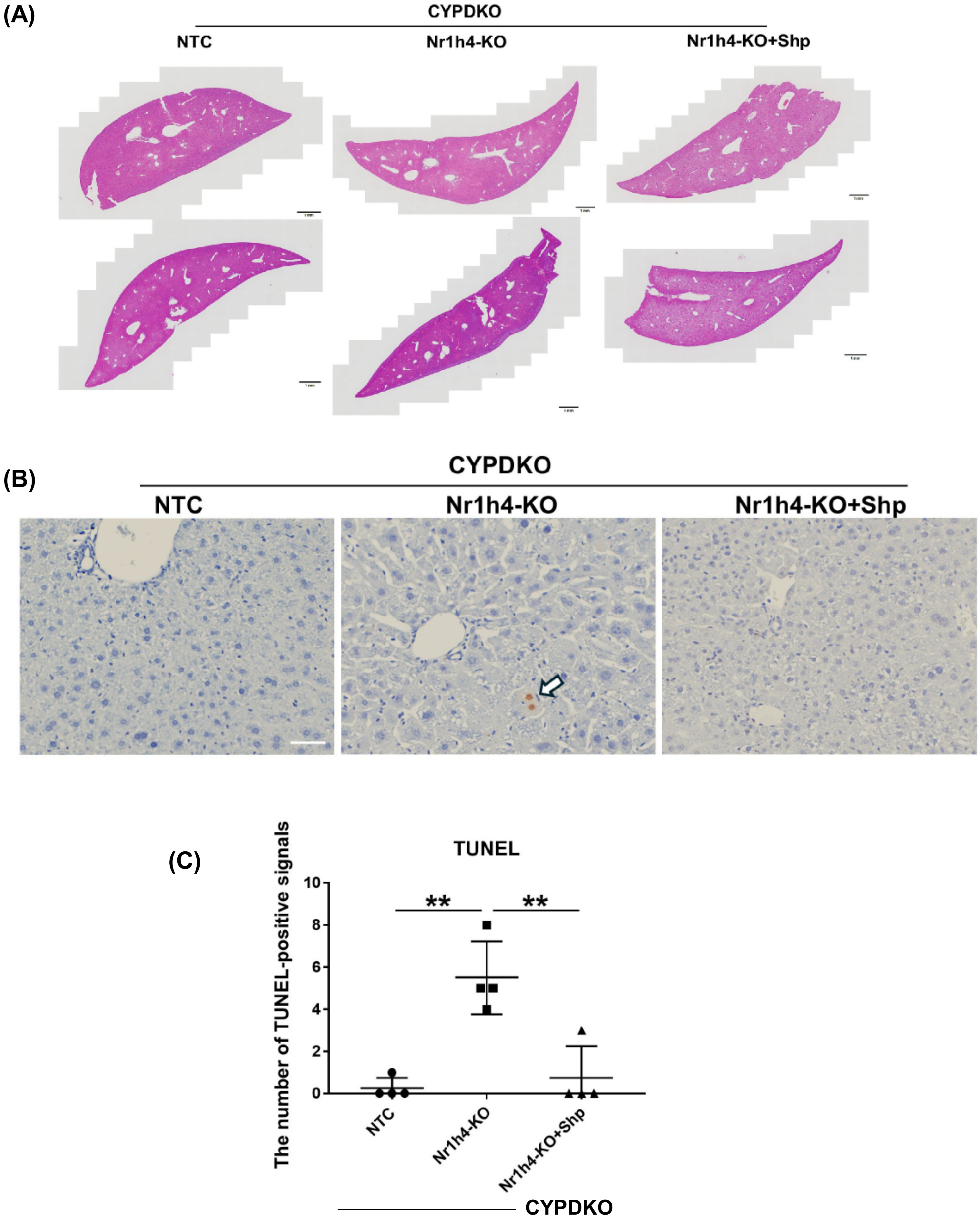

Figure S4 Induction of apoptosis in CYPDKO/Nr1h4-KO mice. (A) Whole liver hematoxylin and eosin staining were performed. Black line, 1 mm. (B) TUNEL-positive cells (white arrow) in livers of CYPDKO/NTC, CYPDKO/Nr1h4-KO, and CYPDKO/Nr1h4-KO + Shp mice. White line, 100 μm. (C) TUNEL signal staining was used to quantify the number of apoptotic cells using ImageJ software (n = 4 for CYPDKO/NTC, CYPDKO/Nr1h4-KO, and CYPDKO/Nr1h4-KO+Shp mouse livers). Results are represented as mean ± SD (one-way ANOVA, $^{**}P < 0.01$.)

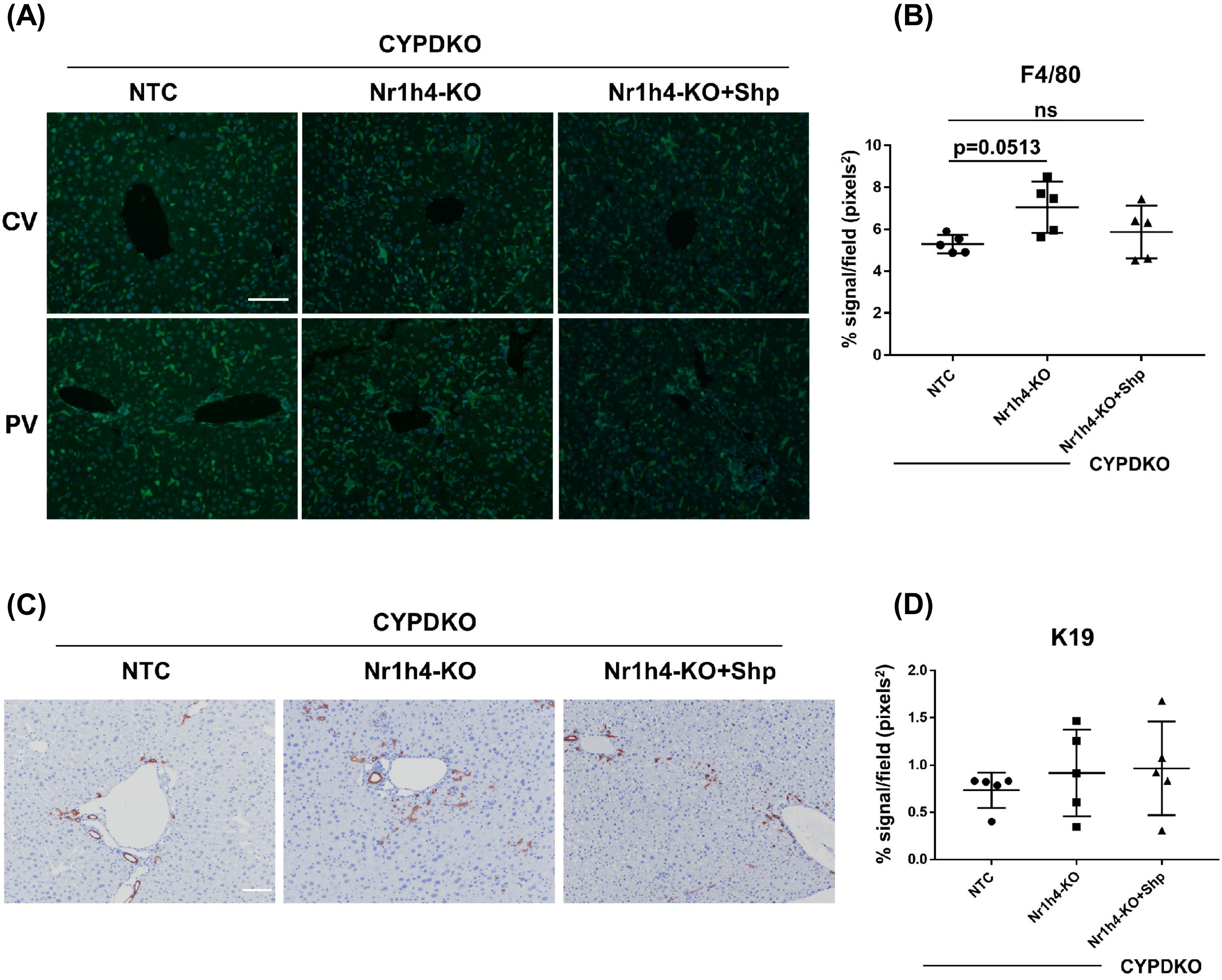

Figure S5 Macrophages and bile duct cells in CYPDKO/Nr1h4-KO mouse livers with Shp overexpression. (A) Expression of F4/80, a macrophage marker, in CYPDKO/Nr1h4-KO mouse liver. F4/80 immunostaining of CYPDKO mice were performed. Nuclei were stained with DAPI. (B) F4/80 staining quantified the number of macrophages using ImageJ software (n = 5 for CYPDKO/NTC, CYPDKO/Nr1h4-KO, and CYPDKO/Nr1h4-KO+Shp mouse livers). (C) K19 immunostaining of CYPDKO/Nr1h4-KO mice with and without Shp overexpression. Nuclei were stained with hematoxylin. (D) K19 staining quantified the number of bile ductal cells using ImageJ software (n = 5 for CYPDKO/NTC, CYPDKO/Nr1h4-KO, and CYPDKO/Nr1h4-KO+Shp mouse livers). CV, central vein; PV, portal vein. White line, 100 μm. Results are presented as mean ± SD (one-way ANOVA).

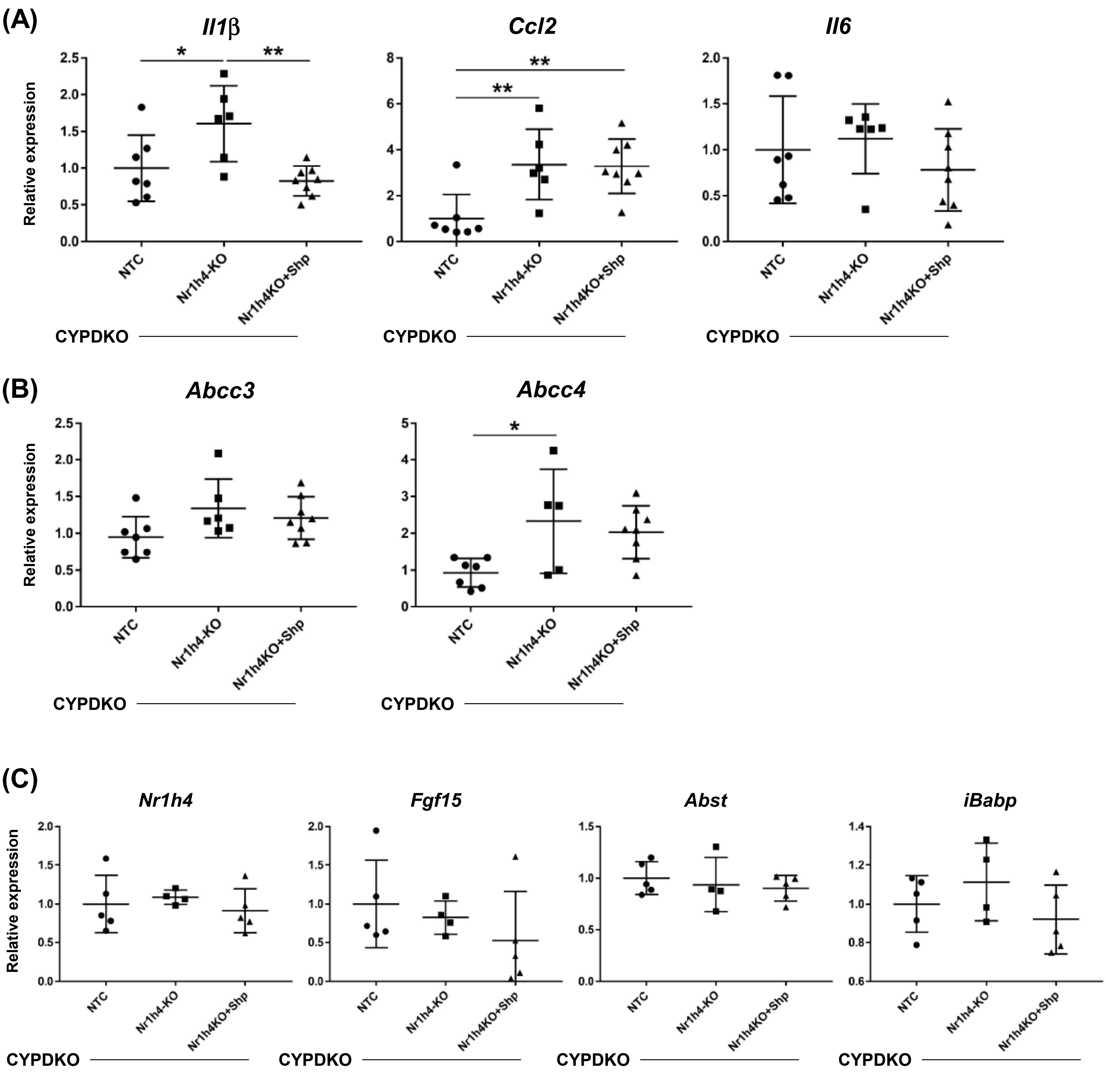

Figure S6 Gene expression in the liver and small intestine of CYPDKO/Nr1h4-KO mice. (A-B) Gene expression changes in inflammatory cytokines (A) and bile transporter genes (B) in the liver (n = 7 for CYPDKO/NTC, n = 6 for CYPDKO/Nr1h4-KO, and n = 8 for CYPDKO/Nr1h4-KO+Shp). (C) Changes in gene expression in the small intestine. (n = 5 for CYPDKO/NTC, n = 4 for CYPDKO/Nr1h4-KO, and n = 5 for CYPDKO/Nr1h4-KO+Shp). The expression of genes CYPDKO/NTC mice was set to 1.0. Results are presented as the mean ± SD (one-way ANOVA, *$P < 0.05$, **$P < 0.01$).

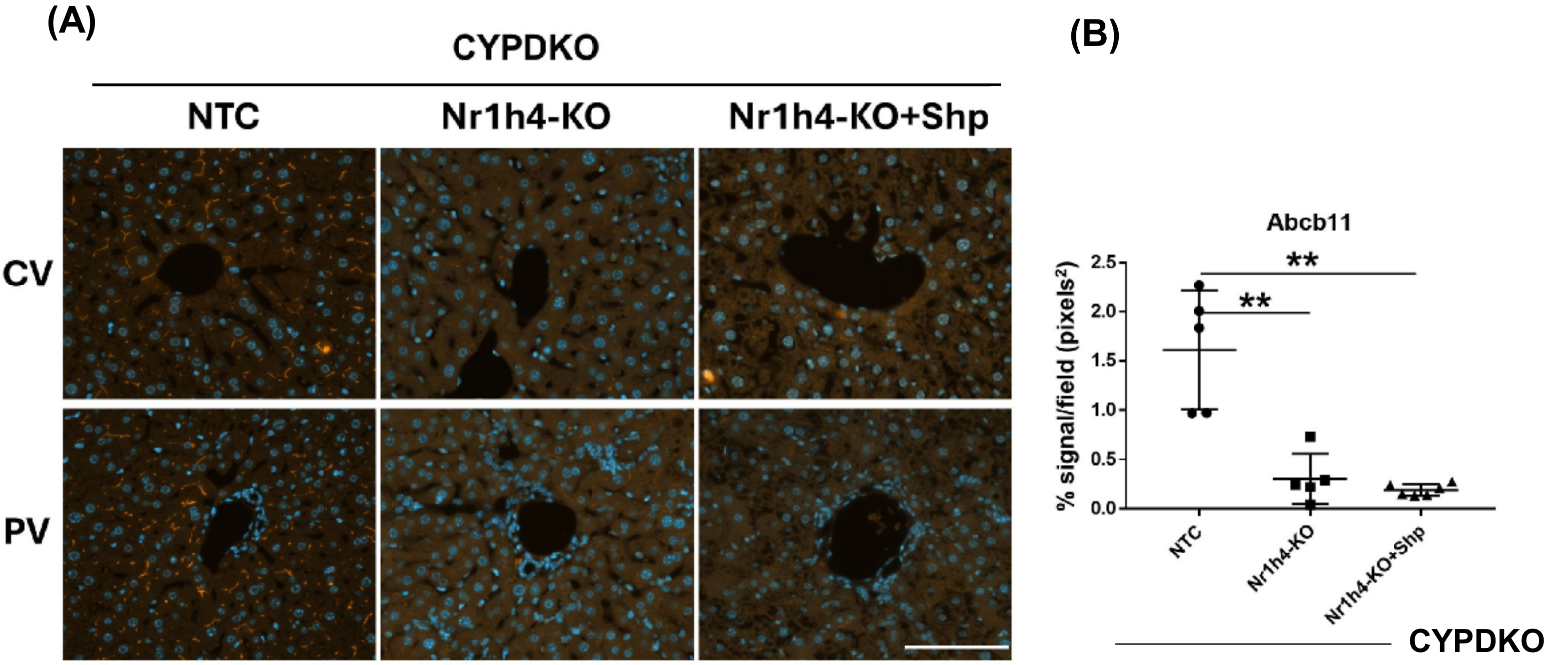

Figure S7 Bile acid transporter expressions in Nr1h4 deficient mice. (A) Expression of Abcb11 (red) in CYPDKO mice. Nuclei were stained with DAPI (blue). (C) Abcb11 staining was used to quantify bsep protein production using the ImageJ software (n = 5 for CYPDKO/NTC, CYPDKO/Nr1h4-KO, and CYPDKO/Nr1h4-KO+shp mouse livers). CV, central vein; PV, portal vein. White line, 100 µm. Results are represented as mean ± SD (one-way ANOVA, $**P <$ 0.01.)

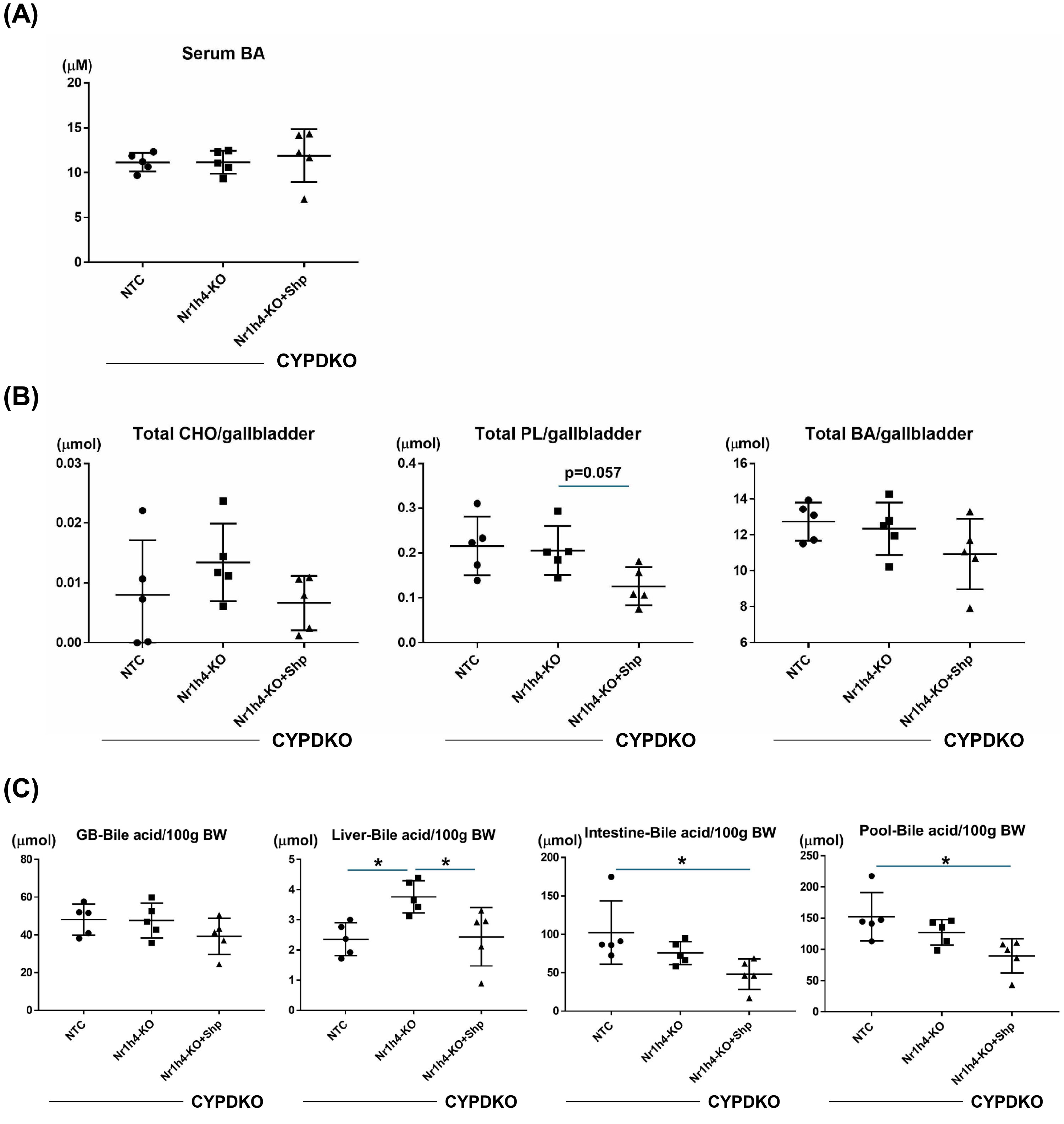

Figure S8 Levels of bile acid and other bile components in the serum and gallbladder of CYPDKO/Nr1h4-deficient mice. (A) Changes in the serum total bile acid levels. (B) Changes in cholesterol (CHO), phospholipid (PL), and total bile acid (BA) levels in gallbladder bile. (C) Bile acid levels of the gallbladder (GB), liver, small intestine, and total pool. BW is body weight. n = 5 for the CYPDKO/NTC, CYPDKO/Nr1h4-KO, and CYPDKO/Nr1h4-KO+Shp groups. Results are presented as mean ± SD (one-way ANOVA, *P <0.05).
