# Supplementary material for: Liver-specific Nr1h4 deletion in mice with human-like bile acid composition causes severe liver injury
Source: J Lipid Res. 2025 Jun 9;66(7):100839. doi: 10.1016/j.jlr.2025.100839 (PMC12332403; doi:10.1016/j.jlr.2025.100839)
Supplement: Supplementary Material 2 [file mmc2.pdf]

Supporting Materials

Liver-specific Nr1h4 deletion in mice with human-like bile acid composition causes severe liver injury

Yusuke Mishima, Kota Tsuruya, Kinuyo Ida, Satsuki Ieda, Yutaka Inagaki, Akira Honda, Tatehiro Kagawa, and Akihide Kamiya

Supplementary Materials and Methods

Serum physiological marker analyses

After anesthesia, blood was collected from mouse hearts. Serum was separated using Bloodsepar (ImmunoBiological Laboratories Co. Ltd., Gunma, Japan). The levels of total cholesterol, HDL, triglycerides, aspartate aminotransferase (AST), alanine aminotransferase (ALT), alkaline phosphatase (ALP), and total bilirubin were measured using the Spotchem EZ (Arkray. Inc, Kyoto, Japan). The measurement ranges for each compound were as follows: total cholesterol, 50–400 mg/dL; HDL, 10–150 mg/dL; triglycerides, 25–500 mg/dL; AST, ALT, and ALP, 10–1000 IU/L; total bilirubin, 0.4–1.5 mg/dL. Values below the detection limit were defined as the lowest values.

Western blot analyses

After collection, the liver tissues were immediately frozen in liquid nitrogen. Nuclear proteins were extracted using NE-PER Nuclear and Cytoplasmic Extraction Reagents (Pierce Bio Inc., Waltham, MA, USA). Protein concentrations were determined using a bicinchoninic acid protein assay kit (Pierce Bio Inc.). Ten micrograms of nuclear protein were mixed with Sodium Dodecyl Sulfate sample loading buffer, and the samples were electrophoresed using SuperSep Ace 10% gel (FUJIFILM Wako Pure Chemical, Tokyo, Japan) and transferred onto an Immobilon-P

membrane (Millipore, Billerica, MS, USA). The membranes were blocked overnight with Block ACE (Waken Btech, Kyoto, Japan) and 5% Tween 20 in TBS (TBS-T), followed by incubation with either anti-Nr1h4 (sc-25309, Santa Cruz Bio, Dallas, TX, USA) antibody at a 1:500 dilution or anti-lamin B1 (12987-1-AP; Proteintech, Rosemont, IL, USA) antibody at a 1:2000 dilution. Membranes were washed with TBS-T and incubated with horseradish peroxidase (HRP)-conjugated secondary antibodies for 1 hour at room temperature. The membrane was washed with TBS-T, and proteins were detected using EZWestLumi plus (ATTO Co., Tokyo, Japan). Sample images were acquired using FUSION SOLO (Vilber, France).

Histological analysis

Histological analyses of the liver injury model have been previously described. Briefly, livers were fixed in 4% paraformaldehyde (FUJIFILM Wako Pure Chemical) overnight and embedded in paraffin. Paraffin-embedded sections were analyzed using standard protocols such as hematoxylin-eosin and Sirius red staining. After each staining, pathological specimens were observed, and images were captured using a BX63 microscope (Olympus, Tokyo, Japan). The degree of fibrosis was calculated using the image sections stained with Sirius Red. Five representative images were collected from each mouse liver section, and positive signals were quantified using the ImageJ software (Bethesda, MA, USA).

Immunohistochemical staining was performed to detect keratin 19 (K19) and F4/80 in the bile ductal and macrophages. Immunohistochemical staining was performed to detect proliferating cell nuclear antigen (PCNA) in liver proliferative cells. Immunohistochemical staining was also performed to detect ABCB11-positive hepatocytes. Paraffin-embedded sections were heated at 110 °C for 10 min in the DAKO target retrieval solution (Agilent Technologies, Inc., Santa Clara, CA, USA). For K19, PCNA, and ABCB11 staining, low pH retrieval solution was used. For F4/80

staining, high pH retrieval solution was used. After blocking with 5% goat or donkey serum/ Phosphate Buffered Saline, sections were incubated with rabbit anti-K19 (provided by Prof. Miyajima, University of Tokyo), rabbit anti-F4/80 (29414-1-AP, Proteintech), rabbit anti-PCNA (10205-2-AP, Proteintech), or rabbit anti-ABCB11 antibodies (18990-1-AP, Proteintech) overnight at 4 °C. For K19 staining, sections were incubated with HRP-conjugated anti-rabbit IgG antibody (N9340, Merck KGaA, Darmstadt, Germany) for 60 min at room temperature. After washing steps, signals were detected using DAKO Liquid DAB+ Substrate Chromogen System (K3468, Agilent Technologies, Inc.). The positive signal was observed using a BX63 microscope. Five representative images were collected from each mouse liver section, and positive signals were quantified using the ImageJ software. For PCNA and ABCB11 staining, the sections were incubated with Daylight594 anti-rabbit IgG (BioLegend, San Diego, CA, USA) for 60 min at room temperature. For F4/80 staining, the sections were incubated with HRP-conjugated anti-rabbit antibody (Histofine Simple Stain Mouse MAX-PO 414341, Nichirei biosciences Inc., Tokyo, Japan). In addition, the signals were amplified using TSA Plus Fluorescence Systems (Akoya Biosciences, Marlborough, MA, USA) for tyramide signal amplification. After washing steps, the sections were stained with 4',6-diamidino-2-phenylindole (DAPI). The fluorescence was observed using an Axio Imager M2 microscope (ZEISS, Oberkochen, Germany). For PCNA and ABCB11 staining, five representative images were collected from each mouse liver section. For F4/80 staining, three representative portal vein and three representative central vein around images were collected for each mouse liver section. Positive signals were quantified using the ImageJ software.

TUNEL Assay was used to detect apoptotic cells in liver sections. Paraffin sections were analyzed using an in situ Apoptosis Detection Kit (Takara) according to the manufacturer’s protocol. The number of positive cells was determined using a BX63 microscope.

Supplementary Figure legends

Figure S1 Expression of FXR protein regulated by AAV-mediated gene editing. After 4–5 weeks of AAV infection, the livers were analyzed by western blotting. The arrow shows the FXR protein bands and the asterisk shows nonspecific protein bands.

Figure S2 Bile duct cells in CYPDKO/Nr1h4-KO mouse livers. K19 immunostaining of CYPDKO and wild-type mice were performed. Nuclei were stained with hematoxylin. White line, 100 μm.

Figure S3 Bile acid metabolic gene expression changed by Nr1h4 knockout in the CYPDKO background. Gene expression changes in bile acid synthesis enzyme genes in the liver (n = 6 for CYPDKO/NTC and n = 6 for CYPDKO/Nr1h4-KO). The expression of CYPDKO/NTC in mice was set to 1.0. Results are presented as mean ± SD (unpaired *t* test, $^{**}P < 0.01$).

Figure S4 Induction of apoptosis in CYPDKO/Nr1h4-KO mice. (A) Whole liver hematoxylin and eosin staining were performed. Black line, 1 mm. (B) TUNEL-positive cells (white arrow) in livers of CYPDKO/NTC, CYPDKO/Nr1h4-KO, and CYPDKO/Nr1h4-KO + Shp mice. White line, 100 μm. (C) TUNEL signal staining was used to quantify the number of apoptotic cells using ImageJ software (n = 4 for CYPDKO/NTC, CYPDKO/Nr1h4-KO, and CYPDKO/Nr1h4-KO+Shp mouse livers). Results are represented as mean ± SD (one-way ANOVA, $^{**}P < 0.01$.)

Figure S5 Macrophages and bile duct cells in CYPDKO/Nr1h4-KO mouse livers with Shp overexpression. (A) Expression of F4/80, a macrophage marker, in CYPDKO/Nr1h4-KO mouse liver. F4/80 immunostaining of CYPDKO mice were performed. Nuclei were stained with DAPI. (B) F4/80 staining quantified the number of macrophages using ImageJ software (n = 5 for CYPDKO/NTC,

CYPDKO/Nr1h4-KO, and CYPDKO/Nr1h4-KO+Shp mouse livers). (C) K19 immunostaining of CYPDKO/Nr1h4-KO mice with and without Shp overexpression. Nuclei were stained with hematoxylin. (D) K19 staining quantified the number of bile ductal cells using ImageJ software (n = 5 for CYPDKO/NTC, CYPDKO/Nr1h4-KO, and CYPDKO/Nr1h4-KO+Shp mouse livers). CV, central vein; PV, portal vein. White line, 100 μm. Results are presented as mean ± SD (one-way ANOVA).

Figure S6 Gene expression in the liver and small intestine of CYPDKO/Nr1h4-KO mice. (A-B) Gene expression changes in inflammatory cytokines (A) and bile transporter genes (B) in the liver (n = 7 for CYPDKO/NTC, n = 6 for CYPDKO/Nr1h4-KO, and n = 8 for CYPDKO/Nr1h4-KO+Shp). (C) Changes in gene expression in the small intestine. (n = 5 for CYPDKO/NTC, n = 4 for CYPDKO/Nr1h4-KO, and n = 5 for CYPDKO/Nr1h4-KO+Shp). The expression of genes CYPDKO/NTC mice was set to 1.0. Results are presented as the mean ± SD (one-way ANOVA, $^{*}P < 0.05$, $^{**}P < 0.01$).

Figure S7 Bile acid transporter expressions in Nr1h4 deficient mice. (A) Expression of Abcb11 (red) in CYPDKO mice. Nuclei were stained with DAPI (blue). (C) Abcb11 staining was used to quantify bsep protein production using the ImageJ software (n = 5 for CYPDKO/NTC, CYPDKO/Nr1h4-KO, and CYPDKO/Nr1h4-KO+shp mouse livers). CV, central vein; PV, portal vein. White line, 100 μm. Results are represented as mean ± SD (one-way ANOVA, $^{**}P < 0.01$.)

Figure S8 Levels of bile acid and other bile components in the serum and gallbladder of CYPDKO/Nr1h4-deficient mice. (A) Changes in the serum total bile acid levels. (B) Changes in cholesterol (CHO), phospholipid (PL), and total bile acid (BA) levels in gallbladder bile. (C) Bile acid

levels of the gallbladder (GB), liver, small intestine, and total pool. BW is body weight. n = 5 for the CYPDKO/NTC, CYPDKO/Nr1h4-KO, and CYPDKO/Nr1h4-KO+Shp groups. Results are presented as mean ± SD (one-way ANOVA, *P<0.05).

Table S1 CRISPR/Cas9 target sequences

gRNA for the target gene	Target sequence (without PAM)
EGFP-gRNA	AAGTTCATCTGCACCACCGGC
Nr1h4-gRNA1	AATGGCCGCGGCATCGGCAGG
Nr1h4-gRNA2	ACGGCAGACCAACAGACCCTC
Nr1h4-gRNA3	TGTGAGCAGAGCGTACTCCTC

Table S2 PCR primers for detection of mouse gene expression

Mouse genes	Forward primer (5'→3')	Reverse primer (5'→3')	Probe number
Tbp	ggcggtttggctaggttt	gggttatcttcacacaccatga	107
Nr1h4	caaaatgactcaggaggagtacg	tccttgatgtattgtctgtctgg	100
Tnfα	tcttctcattcctgcttgtgg	ggtctgggccatagaactga	49
Tgfβ1	tggagcaacatgtggaactc	gtcagcagccggttacca	72
Col1a1	acctaagggtaccgctgga	tccagcttctccatctttgc	19
Timp1	gcaaagagctttctcaaagacc	agggatagataaacagggaaacact	76
Cftr	cagcagctcaaacaactgga	tgtcacaaggtgggtgaaaa	51
Abcb4	gaggtgaagaagggccagac	ctggaccactgtgctcttcc	74
Abcb11	gccacagcaatttgacaccc	ctaccctttgcttctgccca	63
Ntcp	aaggccacactatgtaccctacgtc	gatgctgttgcccacattga	Syber
Abcc2(Mrp2)	actggacaagccacaattcc	ctgcaggagtgctcgtatca	Syber
Abcc3	atcacagccagttcaaagcca	gctcctctccgaggtgcttt	Syber
Abcc4	gtgcacaccgaggtgaaacc	ggcaggagcaaggtctcttaaa	Syber
Abcb1a(Mdr1a)	tagccaacatagcgcgttcc	gttaatgtgtgcgtgtgtgtgc	Syber
Hmgcr	caccatgccatcgatagaga	gctccttgaacacctagcatct	77
Srebp1c	ggttttgaacgacatcgaaga	cgggaagtcactgtcttggt	78
Shp	ctgaagggcacgatcctct	gcctcctgttgcaggtgt	51
Cyp7a1	tcaagcaaacaccattcctg	ggctgctttcattgcttca	50
Cyp7b1	aattggacagcttggtctgc	ttctcggatgatgctggagt	99
Cyp8b1	caggaagttccgtcgatttg	ggccccagtagggagtagac	60
Cyp27a1	gcctcacctatgggatcttca	tcaaagcctgacgcagatg	Syber
Il1β	agttgacggaccccaaaag	agctggatgctctcatcagg	38
Ccl2	aggtccctgtcatgcttctg	tctggacccattccttcttg	Syber
Il6	gctaccaaactggatataatcagga	ccaggtagctatggtactccagaa	6
Fgf15	gctctgaagacgattgccatc	gtagcctaaacagtccatttcctc	TAMRA-FAM
Abst	tatgggttgctgccctgga	gtgtggagcaagtggtcatgcta	Syber
iBabp	gaggtcgtgggtgacaagtt	ttgcttacgcgctcataggt	22

Table S3 liver bile acid composition

Mouse		free CA	free HCA	free CDCA	free DCA	free LCA	free UDCA	free MDCA	free HDCA	free 7oxo-LCA	free 12oxo-LCA	free 12oxo-CDCA
		nmol/whole liver	nmol/whole liver	nmol/whole liver	nmol/whole liver	nmol/whole liver	nmol/whole liver	nmol/whole liver	nmol/whole liver	nmol/whole liver	nmol/whole liver	nmol/whole liver
CYPDKO/NTC	Average	3.016081469	0.020949663	3.172269613	2.89521175	1.719301674	6.096613571	0.860503739	0.238533926	0.213278035	0.193670895	0.121103362
CYPDKO/Nr1h4gRNA	Average	4.678882248	0.181480795	6.100260659	2.830300674	1.935173252	5.757685387	1.123782721	0.491593911	0.214558	0.112236005	0.239438914
CYPDKO/Nr1h4+SHP	Average	6.187381611	0.218109733	3.585559538	2.468870443	1.591184161	4.550485996	0.558355967	0.288553436	0.316443082	0.063037089	0.392837009
CYPDKO/NTC	STDEV	2.997636035	0.01466415	2.184643625	1.472978869	0.896983139	1.490644041	0.337288117	0.149684572	0.072116822	0.134576273	0.052436188
CYPDKO/Nr1h4gRNA	STDEV	1.277872052	0.148145913	2.046422867	1.352245134	0.717815478	3.354803568	0.539513625	0.295362984	0.192765678	0.10204639	0.194096519
CYPDKO/Nr1h4+SHP	STDEV	5.909140533	0.412147273	3.114268316	1.957155067	1.374871176	4.607108745	0.638175055	0.32767344	0.35023392	0.040195875	0.651758945

		free 7oxo-DCA	free 3dehydro-LCA	free 3dehydro-DCA	free 3dehydro-CDCA	free 3dehydro-UDCA	free 3dehydro-CA	free 3epi-CA	free 7epi-CA	free 12epi-CA	free 12epi-DCA	free 3epi-CDCA+DCA	free 3epi-UDCA	free 3epi-LCA	total free BA
		nmol/whole liver	nmol/whole liver	nmol/whole liver	nmol/whole liver	nmol/whole liver	nmol/whole liver	nmol/whole liver	nmol/whole liver	nmol/whole liver	nmol/whole liver	nmol/whole liver	nmol/whole liver	nmol/whole liver	nmol/whole liver
CYPDKO/NTC	Average	1.233680511	0.400424104	0.369905967	0.088296964	0.137426225	0.028617877	0.51559597	0.340616691	2.119178227	0.009561021	0.236861462	0.178995864	0.632555711	24.85578828
CYPDKO/Nr1h4gRNA	Average	1.841536649	0.539483096	0.460578971	0.173328443	0.196822144	0.06632476	0.822182909	0.627024964	3.653637785	0.010106217	0.560648546	0.513318473	1.175052667	34.34628772
CYPDKO/Nr1h4+SHP	Average	2.909441127	0.691014475	1.237703364	0.20611912	0.099489865	0.142239105	0.512045208	0.483841521	4.348153206	0.014965362	0.533549831	0.227737735	0.87023709	32.53563552
CYPDKO/NTC	STDEV	0.708549713	0.302745104	0.207124319	0.017375936	0.076138815	0.014400095	0.708070703	0.211831126	1.834503886	0.007837369	0.153688908	0.098652439	0.367578437	11.71631848
CYPDKO/Nr1h4-KO	STDEV	1.662613298	0.405409392	0.215535755	0.097418171	0.167794936	0.035351557	0.812668881	0.301627763	3.505541076	0.006998359	0.321315063	0.298096782	0.720272951	7.685944476
CYPDKO/Nr1h4-KO+Shp	STDEV	2.76182068	0.457132327	0.639403262	0.087228192	0.06453105	0.077130203	0.772858271	0.499716209	7.256973748	0.017642886	0.326716584	0.221660183	0.838365988	22.88743832

		G-CA	G-CDCA	G-DCA	G-LCA	G-UDCA	total G-BA
		nmol/whole liver	nmol/whole liver	nmol/whole liver	nmol/whole liver	nmol/whole liver	nmol/whole liver
CYPDKO/NTC	Average	0.102461781	0.152813381	0.698900202	0.159575294	0.257598112	1.37134877
CYPDKO/Nr1h4-KO	Average	0.254572948	0.190148757	0.40395214	0.106944041	0.075771945	1.03138983
CYPDKO/Nr1h4-KO+Shp	Average	0.263506488	0.135953574	0.161197316	0.07559139	0.04299682	0.679245588
CYPDKO/NTC	STDEV	0.11587269	0.135417271	0.63040306	0.104735034	0.228407854	1.063247717
CYPDKO/Nr1h4-KO	STDEV	0.208805122	0.153659707	0.415217128	0.110314511	0.104641306	0.906800041
CYPDKO/Nr1h4-KO+Shp	STDEV	0.354486053	0.179966575	0.178782681	0.071988036	0.039419014	0.740659634

		T-CA	T-CDCA	T-DCA	T-LCA	T-UDCA	T-HDCA	total T-BA	all BA
		nmol/whole liver	nmol/whole liver	nmol/whole liver	nmol/whole liver	nmol/whole liver	nmol/whole liver	nmol/whole liver	nmol/whole liver
CYPDKO/NTC	Average	28.88637513	132.1143156	238.0552572	50.306177	100.001487	2.38511279	552.3682329	578.5953699
CYPDKO/Nr1h4-KO	Average	137.032627	341.2410645	310.1840277	69.83634373	82.51439715	4.499016001	947.9166449	983.2943225
CYPDKO/Nr1h4-KO+Shp	Average	100.5505187	213.8792997	204.632817	63.48819608	76.74800846	3.155408527	664.3388105	697.5536917
CYPDKO/NTC	STDEV	22.29580237	51.08755503	60.26615415	14.71688971	34.22760006	1.295178076	138.4453254	147.8959702
CYPDKO/Nr1h4-KO	STDEV	104.6521823	104.6819566	64.27295384	19.71025736	33.91382484	2.601550076	198.1722624	197.5237754
CYPDKO/Nr1h4-KO+Shp	STDEV	90.20158807	195.09312	149.066701	45.08466315	51.16356327	2.484628898	484.7602803	499.2777911
